# An informatics model for tissue banks – Lessons learned from the Cooperative Prostate Cancer Tissue Resource

**DOI:** 10.1186/1471-2407-6-120

**Published:** 2006-05-05

**Authors:** Ashokkumar A Patel, John R Gilbertson, Anil V Parwani, Rajiv Dhir, Milton W Datta, Rajnish Gupta, Jules J Berman, Jonathan Melamed, Andre Kajdacsy-Balla, Jan Orenstein, Michael J Becich, the Cooperative Prostate Cancer Tissue Resource

**Affiliations:** 1 Department of Pathology, Center for Pathology Informatics, Benedum Oncology Informatics Center, University of Pittsburgh, Pittsburgh, PA, USA; 2 Departments of Pathology and Urology, Emory University, Atlanta, GA, USA; 3 Cancer Diagnosis Program, National Cancer Institute, National Institutes of Health, Bethesda, MD, USA; 4 Departments of Pathology, New York University, New York, NY, USA; 5 Departments of Pathology, University of Illinois at Chicago, Chicago, IL, USA; 6 Departments of Pathology, George Washington University, Washington, DC, USA

## Abstract

**Background:**

Advances in molecular biology and growing requirements from biomarker validation studies have generated a need for tissue banks to provide quality-controlled tissue samples with standardized clinical annotation. The NCI Cooperative Prostate Cancer Tissue Resource (CPCTR) is a distributed tissue bank that comprises four academic centers and provides thousands of clinically annotated prostate cancer specimens to researchers. Here we describe the CPCTR information management system architecture, common data element (CDE) development, query interfaces, data curation, and quality control.

**Methods:**

Data managers review the medical records to collect and continuously update information for the 145 clinical, pathological and inventorial CDEs that the Resource maintains for each case. An Access-based data entry tool provides de-identification and a standard communication mechanism between each group and a central CPCTR database. Standardized automated quality control audits have been implemented. Centrally, an Oracle database has web interfaces allowing multiple user-types, including the general public, to mine de-identified information from all of the sites with three levels of specificity and granularity as well as to request tissues through a formal letter of intent.

**Results:**

Since July 2003, CPCTR has offered over 6,000 cases (38,000 blocks) of highly characterized prostate cancer biospecimens, including several tissue microarrays (TMA). The Resource developed a website with interfaces for the general public as well as researchers and internal members. These user groups have utilized the web-tools for public query of summary data on the cases that were available, to prepare requests, and to receive tissues. As of December 2005, the Resource received over 130 tissue requests, of which 45 have been reviewed, approved and filled. Additionally, the Resource implemented the TMA Data Exchange Specification in its TMA program and created a computer program for calculating PSA recurrence.

**Conclusion:**

Building a biorepository infrastructure that meets today's research needs involves time and input of many individuals from diverse disciplines. The CPCTR can provide large volumes of carefully annotated prostate tissue for research initiatives such as Specialized Programs of Research Excellence (SPOREs) and for biomarker validation studies and its experience can help development of collaborative, large scale, virtual tissue banks in other organ systems.

## Background

Recent advances in genomic and proteomic research focused on the identification of new cancer biomarkers have led to an emphasis on biomarker validation through translational studies. These types of studies have generated a need for biorepositories capable of providing large numbers of quality-controlled tissue samples with extensive, standardized clinical annotation. Much of the success of these tissue banks will depend on the implementation of a well designed informatics architecture that complements and executes many of the activities of the biorepository [1]. Informatics as a discipline is the collection, classification, storage, retrieval, and dissemination of recorded knowledge [2]. In particular, the development of tissue banking informatics over the last decade has been recognized [1,3-6] as a necessary component for the implementation of broad scale translational cancer research. However, it has only been in recent years that there has been a realization of the need for a technologically advanced tissue banking informatics infrastructure – one that encompasses all aspects of biorepository management – and which is central to the development of translational research programs. The recent literature describing "best practices" in tissue banks has addressed the importance of informatics in biorepositories [7-11].

The NCI Cooperative Prostate Cancer Tissue Resource (CPCTR), also referred as the Resource in this manuscript, is a progressive project that has previously discussed formative and infrastructural issues (i.e. organization issues, procurement issues, IRB issues) related to its initial activities for marketing the large number of annotated prostate cancer specimens to potential investigators [12]. The group's work describing the process of developing common data elements (CDEs) for prostate cancer tissues laid the foundation for much of the Resource's central database [13]. In addition, by demonstrating how to implement the open access Tissue Microarray Data Exchange Specification [14,15] allowed the Resource to share and merge data with other tissue microarray (TMA) files or link to data contained in external biological databases. The current paper builds on those infrastructural components to create a functional enterprise that collects annotated tissues in a manner that complies with statute and with best practice guidelines, organizes the data in a publicly accessible database, provides a user interface for the public to interrogate the database, permits web-based requests for tissues, provides a fair review process, delivers tissues to scientists, permits growth and continual updates and integration with other ongoing efforts, and markets the entire process. Here we describe all the components that make up this comprehensive CPCTR informatics architecture and look back at lessons learned that contain generalizable observations that other similar biorepository projects can build upon the experiences of the Resource.

## Methods

### Participating institutions

The Resource comprises four academic institutions: George Washington University Medical Center (GWU), Washington, DC; Medical College of Wisconsin (MCW), Milwaukee, WI; New York University School of Medicine (NYU), New York, NY; and the University of Pittsburgh (PITT), PA. The Resource has access to frozen tissues, paraffin blocks, tissue microarrays (TMAs) and a variety of fluids. Cases have between five and ten years of follow up data, and are collected from a variety of medical care settings that include university medical centers, as well as private, public, and Veterans Administration hospitals. The participating hospitals are distributed across six states in the Northeastern and Midwestern regions of the USA. This varied access to cases allows accrual of material that reflects a wide diversity of patients undergoing prostate cancer management in the United States.

### Criteria for inclusion of cases into Resource

All prostate cancer patients are registered in the CPCTR if there is at least 5 years of clinical follow-up (except for frozen specimens), if they have at least one Matrix Block containing tumor, and if the critical Clinical Data Elements (CDEs) are completed. In cases with only a single Matrix Block containing only a small amount of tumor, the specimen may not be available for distribution, owing to the need to preserve the diagnostic tissue in the Surgical Pathology files. The follow-up information required for the patient's file would include vital status and PSA values for as many years as possible.

### Human subjects protection – The honest broker concept

The CPCTR collects tissue and data locally. Tissue is stored locally but data management is done centrally. Each member institute has developed its own local protocols including consent language describing its procedures to protect the confidentiality and privacy of human subjects and has obtained local IRB approval for all CPCTR activities. In each case the individual patient consents have been written as broad tissue banking protocols, thus ensuring a uniform approval for the use of the specimens in multiple types of research studies.

The institutions that make up the Resource ensure protection of patient identity through "The Honest Broker Concept." An "honest broker" or "tissue bank trustee" acts as a well defined barrier between the clinical environment (in which fully identified confidential patient information is routinely exchanged as part of medical care) and the general research community (in which all information must be completely de-identified). In its purest form, the honest broker is not part of either the clinical or research team and is the only person or organization that can link research identifiers and clinical identifiers. In most cases, provisions are in place for tumor registrars at the local CPCTR site to act as the honest broker. By using the honest brokers one has placed control and responsibility of the de-identification process in the hands of an independent third party, reducing the risk of conflict of interest. Personal and clinical identifiers (names, medical record numbers, etc.) are limited to the clinical space while research identifiers (i.e. "subject 12432") are never tied to the personal or clinical identifies except through the honest broker's code book. This concept differs from anonymization, which is a one-way process of removing the linkage between personal identifiers with research identifiers and which does not allow for subsequent updating of the data.

This concept is implemented by having at least one tissue bank trustee acting as the honest broker at each institution. It has become extremely valuable to have a tumor registrar act as a designated tissue bank trustee. Tumor registrars, by the nature of their job and by federal mandate already have access to clinical information on cancer patients, yet they do not have access to the results of research data for tissue bank samples. The trustee is the only person who can link a patient with the tissue bank number that identifies that patient. The trustee system ensures that new clinical outcome information can be added to a file identified only by a code number, not a name. Additionally, in the extremely rare event that important research data becomes available and it becomes necessary to inform the patient or their survivors, a fail-safe mechanism through the tumor registrar exists for such information to reach the interested party.

### Development of the common data elements (CDE)

By collaborative consensus the CPCTR CDE subcommittee developed 145 data elements to annotate the tissue samples that have been collected. For each case, these include: 1) patient-level demographic and clinical history data, 2) pathology specimen-level elements to describe the TNM staging, grading and other characteristics of individual surgical pathology cases, 3) tissue block-level annotation critical to managing a virtual inventory of cases and facilitating case selection, 4) and patient level clinical outcome data including treatment, biochemical (prostate specific antigen [PSA] values) and clinical recurrence, and vital status. The development and implementation of these CDEs by the CPCTR CDE subcommittee was facilitated by knowledge gained and shared from other groups, including the Cooperative Breast Cancer Tissue Resource CBCTR [16,17] as well as established open source standards from the AJCC Cancer Staging Manual[18], the NAACCR Data Standards for Cancer Registries[19], the CAP Cancer Checklist[20], and other prostate specific CDEs that were available through the NCI Center for Bioinformatics (NCICB) [21,22]. The full description of the process involved with the freely available CDEs developed by the CPCTR has been previously described [13].

### Data collection

Tissue and data collection occurs independently at each site. When a case becomes available, Resource pathologists review the surgical pathology report and all histological sections available to accurately categorize each prostate cancer case. The pathologist then selects (two to five) key slides according to a standardized protocol. The selected slides show specific features of the case (such as high tumor volume) likely to be of interest for scientific investigators. Specific data elements are collected on these slides. These slides, henceforth termed "matrix" slides, are used to retrieve the corresponding "matrix" blocks which represent the core specimen component of the Resource.

Once the pathological data is reviewed, data managers, and certified tumor registrars review and extract clinical data for cases accrued into the Resource. The data are derived in part from the tumor registries of the various hospitals and institutions. Additional in-depth clinical information is obtained by direct review of and extraction of information from patient charts, from consultation with outpatient referring physicians, and from direct patient interviews performed by cancer registrars and clinical nurses. Data is collected and annotated using common data entry paper forms that are correlated with the CDEs developed by the Resource. The data entry paper forms and the CDE data dictionary for both the pathology dataset as well as the clinical follow up dataset are available for downloading as part of a previous manuscript [13].

### Development of data collection applications and common methods for data transmission

Once the initial set of CDEs was developed and approved by the CPCTR Coordinating Committee, it was used to design and create a Microsoft Access data entry application by Information Management Services, Inc. (IMS, Bethesda, MD)[23], which was the central data management site for the Resource. This data entry tool, along with the data entry paper forms, were then distributed to each of the member institutes to allow capture of standardized, structured data on all of the tissue samples they provide to the Resource. The application also included a set of Central Data Center de-identification numbers that were randomly generated and pre-assigned to each institution. These de-identification numbers are attached to individual cases at each institution and are subsequently used as the de-identified code for tissue and data collection and redistribution. The Resource has also designed a CDE data dictionary to provide guidelines for each of the data managers regarding the definition of each CDE, its valid values, variable constraints, validation rules, and any requirements and useful comments with regard to each of the CDEs. It also notes the inclusion criteria of the types of biospecimens collected for the Resource. This data dictionary is a dynamic document that is freely available and is regularly updated and refined as specific information or issues regarding various CDEs arise.

**Figure 1:**
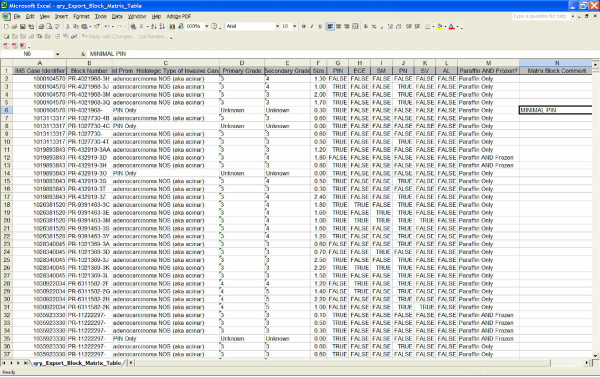
**Example of a pre-defined export file**. An example of one of the pre-defined export file (prostatectomy block matrix file) in Microsoft Excel.

Each member institute has the option to either utilize this Access database or develop their own database based on the technologies present in their institutional environment. Two of the member institutions created their own databases using Oracle, while the two other institutions modified the Access database to collect other data elements unique to their local biospecimen collection efforts and research activities. Every institution was required to transmit only the common de-identified data to the Central Data Center's central database on a monthly basis utilizing pre-defined data files with Microsoft Excel worksheets. An example of one of the pre-defined export files is shown in figure 1. Although dates were represented as month and year when data was exported to the central database, all dates were converted to ranges in months from/to diagnosis date when shared with scientific researchers. Furthermore, all of the HIPAA's proscribed set of 18 data elements types were omitted from the transmitted sample records such that the central IMS database contained only de-identified HIPAA compliant patient data. A similar approach was used in the construction of the web-accessible database for use by the scientific research community for specimen data queries, data analysis, and specimen selection.

### PSA algorithm

Outcomes data related to prostate cancer often utilize surrogate outcomes indicators such as PSA recurrence. While the criteria for PSA nadir and recurrence may differ between studies, a clearly defined and uniformly applied algorithm for calculating PSA results needs to be applied to all the specimens used in a study. The Resource has developed mechanisms for collecting critical pre-treatment PSA values as well as post-surgical PSA values as part of the ongoing follow up of cases. Interpretation of PSA data is performed in a uniform manner at the central data center through an algorithmic assessment and categorization of cases into biochemical recurrence cases, non-recurrence cases, cases with post-surgical residual elevated PSA or cases whose category "cannot be determined". The algorithm's PSA thresholds have been determined through a review of the literature and consultation with prostate cancer experts and were validated at each site through a comprehensive analysis of clinical features of a series of individual cases. The details of this algorithm have been reported elsewhere[24], and provide a uniform reproducible method for PSA outcomes based studies using the Resource's specimens.

### Data quality assurance

#### Quality assurance for data transmission

Once data is imported into the central database, the Central Data Center processes the data using policies, variable constraints, and logistical tests established by the Resource. Quality Assurance (QA) checks are conducted to detect any missing essential CDE data or possible data input errors including field and cross-field checking (i.e. number of nodes positive >1, then pathology nodal stage = pN1). The valid field options, defined for each data element in the CDE dictionary, are checked using these automated QA measures. Accepted records are subsequently loaded into the central database. Unacceptable records are not be loaded into the database and are returned to the submission site for review and correction. The Central Data Center documents the reasons for rejection when the unacceptable records are returned for correction. Furthermore, any records with invalid or discrepant data items are censored (i.e., removed from the available tissue samples for investigators) so they are not selected for an application request until they are resolved. Resolutions are the responsibility of the sending institution and are repaired and re-sent with the next monthly data update.

#### Quality assurance for clinical data

##### Clinical data audit

The large number of CDEs collected by the CPCTR represents a subset of the material collected by cancer registrars through review of in- and out-patient medical records, pathology, radiology, radiation therapy, laboratory reports, etc. The Resource has established an audit review system that seeks to verify the abstracted material by re-review of the primary source clinical information. In this audit review system the Central Data Center selects 10% of the newly entered cases to be examined.

Independent qualified individuals who are not directly involved in the funded Resource are recruited as audit reviews to examine the randomly selected audit review cases and compare the annotated CDE data against the patient's clinical information. The audit reviewers are Physicians, Tumor Registrars, Data Managers, or technicians that are not part of the regular data collection process as well as honest brokers at each of the institutions. Upon completion of their review, the audit reviewers submit a report of their findings and their recommendations to the Resource. The Resource members discuss their findings in the next general meeting of the Resource Coordinating Committee and make plans to implement the proposed recommendations. Each site is responsible for making any necessary corrections discovered during the audit process and submitting their corrected data to IMS. As an extra precaution, before any specimens are sent to investigators, a final review of the associated case CDEs for possible errors and an update of the clinical information is performed to guarantee the most accurate and up-to-date information for requesting investigators.

#### Quality assurance for pathological data

All pathologic CDEs related to the specimen cases are entered after complete specimen review by trained urologic pathologists. In order to standardize this process the Resource has established a histopathology manual [25] for use in the diagnosis and assessment of cases, which acts as the standard reference for Resource pathologists. In addition the CPCTR has periodic QA assessments of pathologic data collection, with a specific emphasis on inter-observer concordance of pathologic review by the Resource pathologists. This process includes: 1) joint review of selected pathologic cases at meetings and 2) the independent review of cases via a) actual physical slides being sent around from each site or b) a web-based microscopy QA protocol using digitalized slides.

For joint QA review during meetings, Resource pathologists review 5 cases from each site, with emphasis on the 5 "matrix" slides. Joint review of cases on a multi-headed microscope permits the Resource pathologists to discuss diagnostic differences and set thresholds. The cases are selected by pathologists at each site to include difficult rare cases (such as rare histologic patterns of tumor or tumors with Gleason grades 2 or 3) or difficult diagnostic cases that illustrate areas of possible disagreement (with emphasis on difficult assignments of Gleason grade, pathologic stage and margin status).

In the Independent review process a series of randomly selected cases are sent between Resource sites for re-review. The Central Data Center randomly selects cases for Independent review from those added to the Resource within certain cut-off dates. The Independent review material consists of 2 to 5 matrix slides for each case. Once received at a Resource site the Resource pathologists review and annotate the pathologic matrix and histology CDE data for the case using their established processes. The completed data fields are then sent to IMS for analysis of inter-observer and intra-observer variability, outlier calculations, and diagnostic error rates. This process occurs at regular intervals (2 times/year) in the Resource history, and is established to check specimen resource quality. As an alternative to the shipment of individual glass slides the Resource has also utilized web-based QA protocols using digitalized slide images. The Independent review slides were sent to a central site for scanning, data storage, uploading to an interactive web interface for the QA evaluation. This reduced the risk of slide loss or damage through multiple shipping sites [26].

Any discrepancies identified through Independent review are communicated by the Central Data Center to the Resource pathologists via the pathology subcommittee. The pathology subcommittee then discusses their findings in the subsequent general meeting of the Coordinating Committee through a formal report with recommendations for changes in process as indicated by the Independent review findings. Each site is subsequently responsible for correcting any errors discovered during the Independent review process and submitting their corrected data to IMS.

### Central data center

In addition to managing the data collection and transmission data system (vide supra), the central data center manages an online query database. The primary purpose of this database is to allow managers and investigators across the CPCTR to query the entire data set online to determine the availability of CPCTR specimens and data for proposed investigator initiated research projects. The central database contains de-identified information about the available tissue blocks along with their associated demographic, pathologic, clinical, and follow up data.

#### The central database

**Figure 2:**
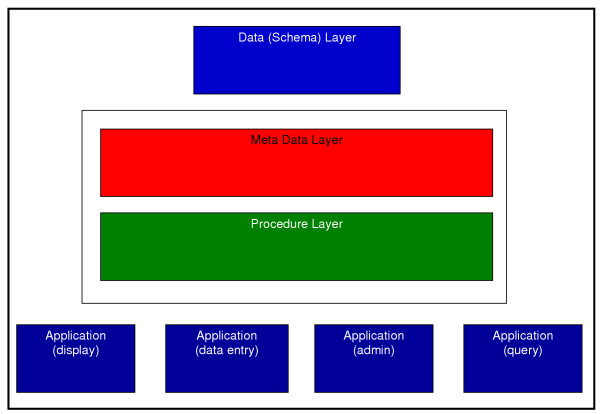
**An overview of the CPCTR system design**. The overall system is designed for the CPCTR central database as a multi-tiered application using Oracle 9i.

The overall system is designed as a multi-tiered application using Oracle 9i as shown in figure 2:

• *Schema layer *– actual data and data relations. All data is stored in numbers and keys.

• *Meta data layer *– in which all data is defined in terms of data elements and "groups of data elements". Data descriptions such as data attributes, display attributes, valid values, DB Link, validation rules and documentation are supported in meta data. The meta data layer defines the application layer.

• *Procedures/function layer *– a set of dynamic procedures/functions (in PL/SQL or Java) with control data transformation at the back end. The procedures accommodate changes in the meta data and immediately reflect the changes in the application layer.

• *Application layer (Form builder) *– a set of "applications" including meta-data dictionary builder and manager, user management, data entry/transfer, query, display, etc. Depending on the user privileges, the appearance will be different. These differences are driven by the meta-data and user management.

#### Data query tools

**Figure 3:**
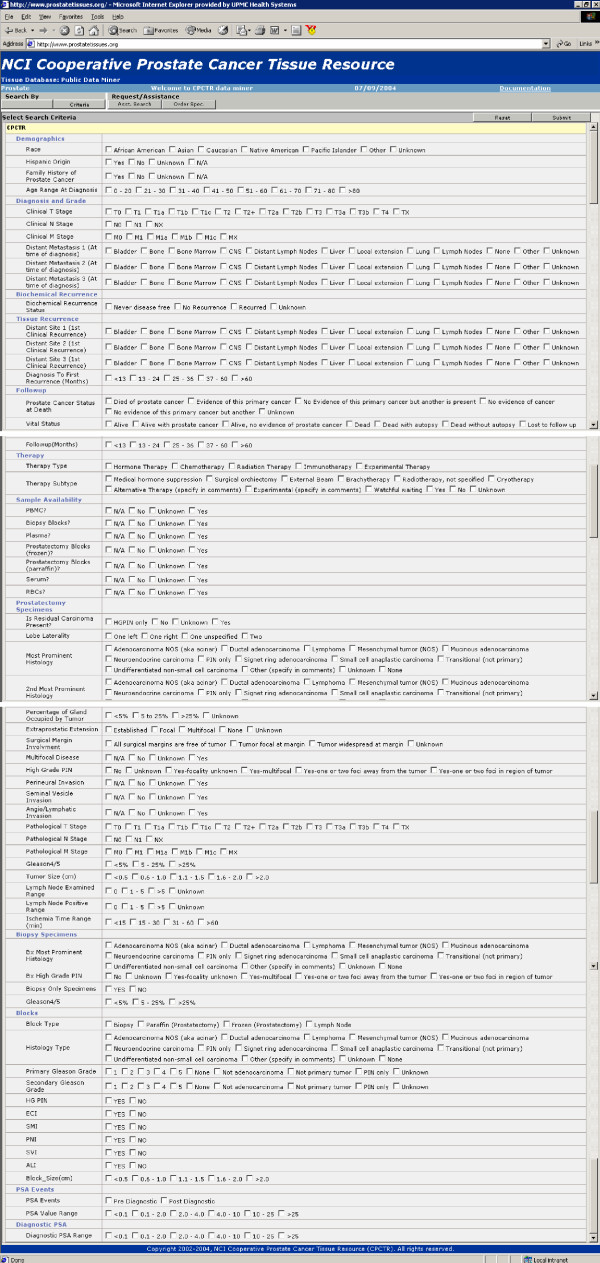
**Criteria selection page**. Users can select the specific criteria for searching the central database.

**Figure 4:**
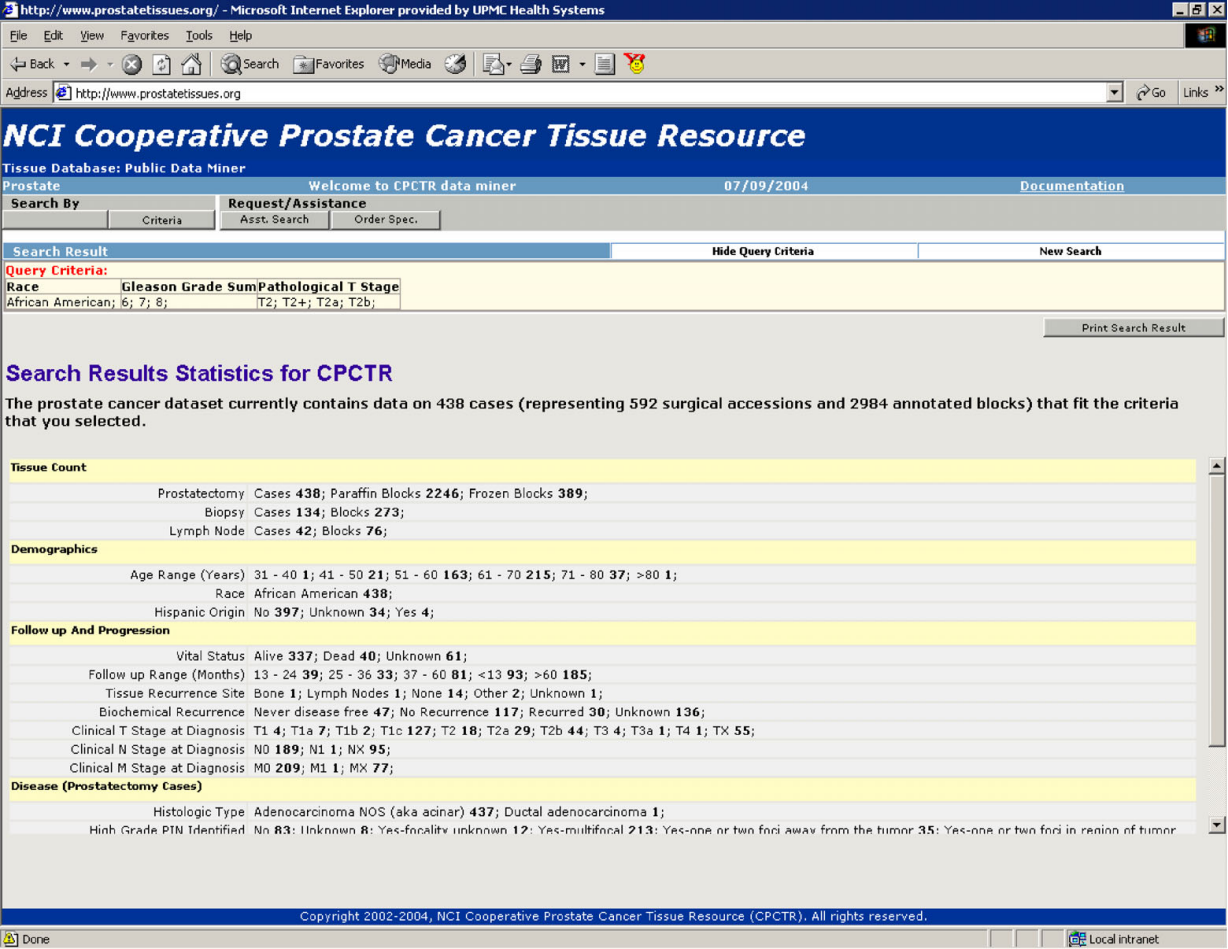
**Public Query Tool**. Public Query Tool showing statistical information on cases available through the CPCTR.

**Figure 5:**
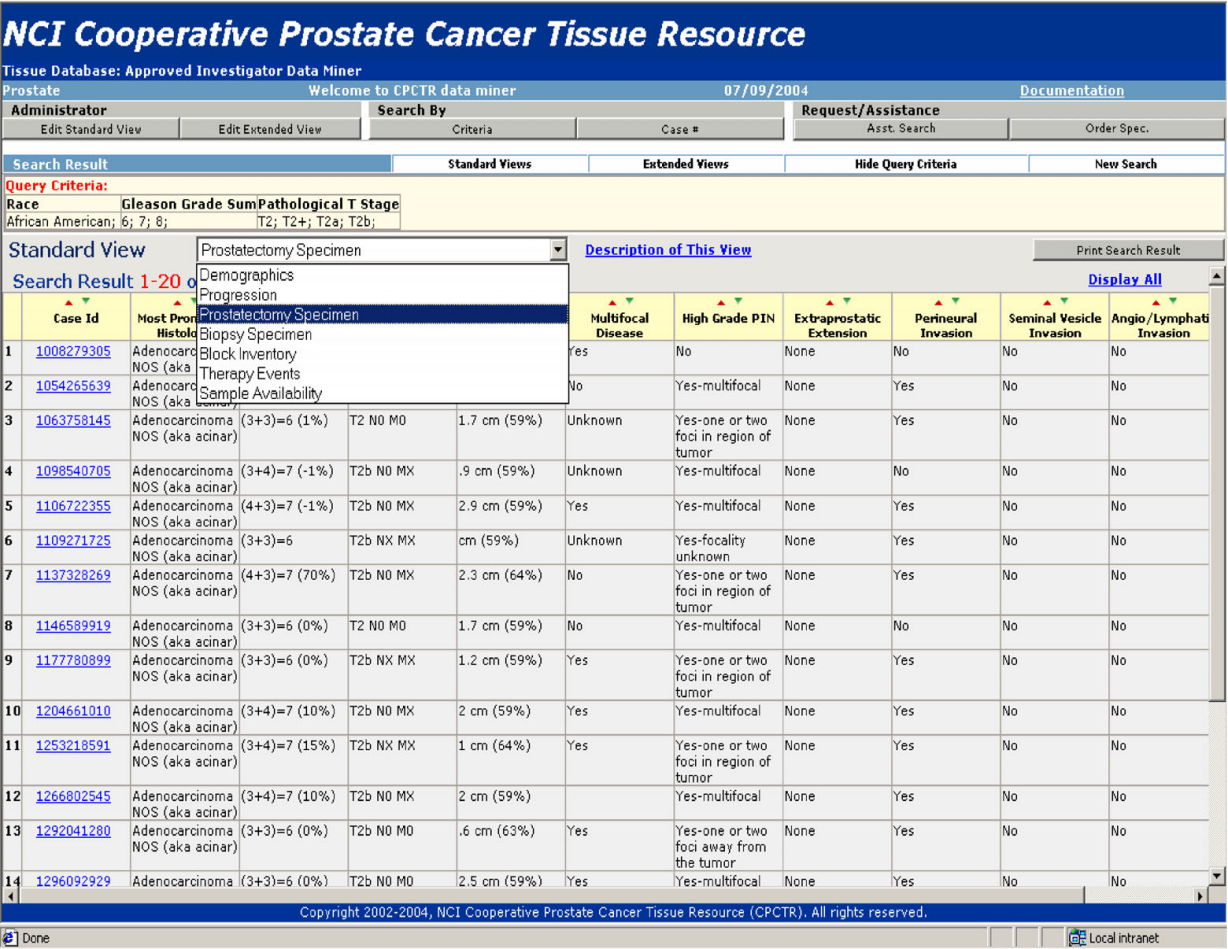
**Approved Investigator Query Tool**. Approved Investigator Query Tool showing detail annotation of cases available. Users of this level can view data in multiple pre-defined views.

**Figure 6:**
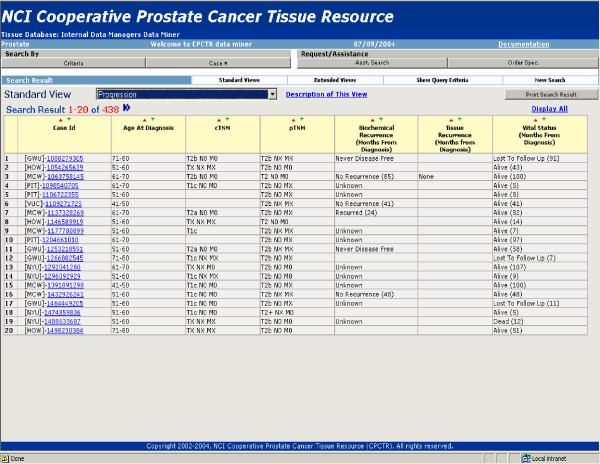
**Data Managers Query Tool**. Data Managers Query Tool is for internal CPCTR members. This view allows users to see which institute submitted each case.

The Central Database's main user interface is through a central query tool. This central query tool uses a 'click and point' interface that allows queries on virtually all 145 data elements shown in figure 3. However, the specificity of the data returned will depend on the user's profile. There are three user profiles as follows:

1. *The public query tool *is utilized by potential investigators as well as open to the general public and is accessible through the Resource's web site [27,28]. The output of a public query tool, as seen in figure 4, is designed to provide would-be investigators with restricted summary information regarding the numbers of specimens in the Resource. This is supplied as the number of cases, specimens and blocks in the database that meet the criteria of the query and general statistics on a limited number of data elements. From these queries interested investigators should be able to derive sufficient information to determine if the Resource has sufficient specimens to meet experimental requirements.

2. *Approved investigator query tool *is secure password protected tool that is distributed to investigators with approved CPCTR protocols. It allows users to refine case lists for their applications and mine the data related to the specimen cases they have received from the Resource for their approved studies. These queries return all de-identified CDE data associated with each approved case through multiple pre-defined views of the data set as shown in figure 5.

3. *Data manager query tool *is a secure password protected tool restricted to the internal CPCTR members. It is meant for data managers and key NCI members to address and review QA results and processes for site-specific and overall Resource data. It also allows senior CPCPR personnel to query the database during the evaluation stage of proposed Resource projects to establish project feasibility. An example would be a query of the central database to identify if sufficient specimens are present to create an ethnicity tissue microarray. The main difference between the Data Manager query tool and the Approved Investigator query Tool is the ability to identify individual submitting Resource institutions for each specimen (i.e. GWU, NYU, MCW, or PITT), for the purposes of generating a tissue disbursement or request lists as shown in figure 6.

Additional activities of the Central Data Center, as directed by the Resource, include: 1) track and respond to investigator inquiries regarding the Resource. 2) process specimen requests and coordinate letters of intent, applications, and specimen shipments. 3) prepare invoices, collect payments, and distribute payments to Resource institutions for shipped specimens. 4) Develop and maintain the central database as well as the Resource website [28], and 5) generate data reports from the Resource central database.

### Marketing of the resource specimens

The CPCTR marketing sub-committee uses various media for advertising the availability of CPCTR tissues and services to the research community. These methods have included, but were not restricted to:

1. *A public website *[28] that has been built and maintained by all sites, with the support from the Central Data Center. The website includes general information about the CPCTR, information about the type of specimens available, a searchable database of the cases (vide supra), and online forms for making tissue requests and inquiries.

2. *Mass mailings *to potential users: Letters and/or e-mails are sent to investigators that have published articles in tissue-based prostate research. Additional names were provided by investigators who visited the CPCTR booth at scientific meetings. A mechanism is provided for any email subscriber to "opt out" from the mailing list at the time any mass mailing is distributed.

3. *Advertisements *are placed in specialty scientific journals, research society newsletters, fliers at research meetings, and through free listings in journals and websites.

4. *Word of mouth*, through individual Resource investigators and their scientific contacts, in particular at scientific research meetings.

5. *Posters and podium presentations *regarding the practical use and the Resource specimens at research meetings.

6. *Marketing booths *at scientific research meetings, in conjunction with other NCI resources, or as individual stand-alone booths.

With an initial budget allocation of approximately $10–15 thousand, the Resource was able to purchase a professional marketing display booth as well as print flyers and bookmarks for distribution. Subsequently, revenue generated from approved tissue requests were divided to each institution based on the number of samples distributed from each site and utilized for further marketing efforts (i.e. print advertisements and scientific meetings). All marketing expenses were shared equally amongst the members. In additional, marketing surveys were conducted at scientific research meetings as well with prominent independent prostate researchers to gauge what resources the CPCTR should focus on providing to the research investigators.

### Implementation of standards

During the development process of the CDEs, open source standards including the AJCC Cancer Staging Manual[18], the NAACCR Data Standards for Cancer Registries[19], the CAP Cancer Checklist[20], and other prostate specific CDEs that were available through the NCI Center for Bioinformatics (NCICB)[21,22] were utilized to create the Resource CDE dataset. The Resource has also implemented an open access Tissue Microarray Data Exchange Standard[14] that has allowed the sharing of the clinical and pathological data associated with Resource developed tissue microarrays that are distributed to investigators[15]. The Resource has put into practice IATA Dangerous Goods Regulations[29] for the special handling and safe shipping of frozen tissues to investigators. This standard is recognized by major courier services when shipping biohazardous materials by air.

### Reference manuals and documents

A Manual of Operations [Additional file 1] was created and used by the Resource that details the governance, protocols and guidelines used to operate a large prostate biorepository with well annotated clinical data. It describes the common practice used by each of the institution for QA/QC protocols, as well as tissue collection and pre- and post-storage sample processing. A histopathology manual [25] was also created to standardize the pathological review across all member institutions.

### Information about the resource

The CPCTR website[28] contains additional details about the Resource, including a frequently asked questions (FAQ) section. Investigators are encouraged to post any additional questions to the listserver of the Resource: ASK-CPCTR-L@NCI.NIH.GOV, which will be responded to by one of the PIs who cover the listserver on a rotating basis.

## Results

### Tissue resource

**Figure 7:**
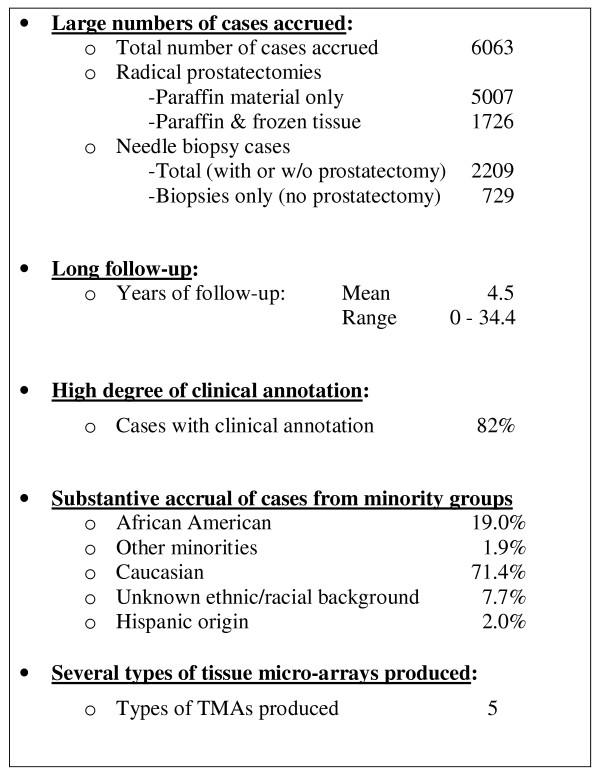
**Summary of the Achievements of the CPCTR**. Summary of the Achievements of the CPCTR

Since July 2003, the CPCTR has offered over 6,000 cases (38,000 blocks) of highly characterized prostate cancer tissue specimens, including tissue microarrays. Median follow-up is 4.5 years and 82% of the radical prostatectomy cases are annotated with follow-up tumor marker data (PSA) critical to predicting recurrence and progression. At the end of its third year in existence (September 2003), the Resource fulfilled its first request for samples and clinical data. This initial use of the resource required highly characterized clinical data, particularly PSA failure data, which was successfully handled by the development of a simple PSA algorithm for biochemical recurrence (vide supra). Figure 7 summarizes some of the accomplishments of the CPCTR in its first 3 1/2 years of operation.

At the time of submission, a total of 131 requests have been submitted as shown in table 1. In 16 cases the material requested was best handled by other NCI funded repositories (CHTN), and the requests were redirected to the appropriate repository. Thirteen requests were either denied by the CPCTR review panel (REP) or were withdrawn by the investigators. An additional 25 were closed by the Resource after failure to obtain response from the investigators (many times due to investigator relocation, project changes, or loss of investigator funding). Of the remaining 77 eligible applications described in table 1, 49 (64%) requests highlighted in green have received specimens for their research, while the remaining 28 requests highlighted in yellow have full or revised applications being completed. The CPCTR has fulfilled 5 of 8 REP-approved requests for large numbers of tissue samples, which has resulted in the distribution of prostate cancer tissue from 440 frozen cases and 200 paraffin embedded cases, with paired serum samples for many of them. In addition, there were 21 REP-approved requests for tissue microarrays (TMAs) with associated clinical data. In several situations, investigators requested small numbers of cases for the generation of pilot data or to demonstrate to the REP that their experimental protocol was suitable for a larger request of materials. These requests were met through a Resource "short-form" process and expedited by approval of the Resource principle investigators. To date 19 such requests have been fulfilled.

### Resource tools and standards utilization

A major portal for investigators to access the Resource is through its public website [28]. Through its web site the Central Data Center has received over 75 email inquires regarding samples available for the research community. As of December 2005, they have tracked the Resource website activity and collected over 6,600 website hits and over 1,800 hits of its Public Query Tool. In addition, the Resource has extended the use of data standards through their implementation and distribution. In particular the Resource has demonstrated how the TMA Data Exchange Specification can be implemented for the CPCTR prostate cancer TMAs [15]. The Resource has also created a program for calculating PSA recurrence using a standardized protocol. With over 39,580 PSA values collected for the 6064 cases in the Resource, the algorithm has categorized: 346 cases into biochemical recurrence cases, 1935 non-recurrence cases, 492 cases with post-surgical residual elevated PSA and 3291 cases whose category "cannot be determined". As additional PSA data is collected these numbers will continue to be updated and improved.

### Consultation of expertise

In efforts to help other collaborative groups who were interested in developing similar projects, the members of the CPCTR have provided consultation services to disseminate their expertise. A short list of institutions to which the Resource has consulted is listed in table 2. The Resource has also shared documents/tools developed within the group, as well as lessons learned and guidance to many barriers that groups might face in developing their tissue banking efforts.

## Discussion

The increased emphasis on cross-disciplinary translational research and the advances in technologies capable of analyzing clinical tissue samples has increased the research community's need for high quality biospecimens associated with a rich set of clinical annotation. Yet potential investigators must be aware and knowledgeable of the specimens, and these specimens must be available through a standardized and equitable process. In order to service these needs, the CPCTR has developed an integrated biorepository and database with accompanying web based query interfaces, and an associated marketing effort. Underlying the biorepository is a rigorous system of common data elements for characterization of tissue samples and clinical follow-up data, supported by a vigorous quality assurance process. Through the central database the CPCTR has developed and implemented a variety of query tools to make investigators knowledgeable of the Resource specimens. Lastly, the Resource has developed efforts to market the specimens, and a process for requesting samples and data by investigators that involves an independent research evaluation panel (REP) of outside experts on prostate cancer, biostatistics, and pathology.

### Annotation – the creation of the local CPCTR dataset

In order to annotate the tissue samples collected for the Resource, the data managers at each of the member institutions manually gathered the relevant clinical information using a variety of data sources. This clinical information was then manually 1) integrated, 2) de-identified, and 3) standardized.

Integration is where a case includes selected patient data from multipleclinical systems over time. An example of this would be merging data from several sources such as:

• Biopsy and resection data on the same patient (largely from the Anatomic Pathology Lab Information System)

• Tumor Marker data (largely from the Clinical Pathology Lab Information System or Medical Record)

• Clinical Staging (largely from the Medical Record or Tumor Registry)

• Treatment data (largely from the Medical Record or Tumor Registry)

Barriers related to this process centered on two issues: 1) identifying the same patient in multiple systems (or hospitals) and 2) identifying the right data within the right context. For tracking outcomes data on patients that utilize multiple health care institutions, one must be able to integrate the patient data from multiple sources. This can be possible through common linking patient identifiers or information, but often can lead to errors based on data entry issues or the lack of common unique patient identifiers. The solution employed by the Resource to address these barriers was largely a manual process of data collection and entry by data managers. In this way the focus was kept on data quality. Yet the derived data still needs to be screened for context before it can be integrated into a database. This is true for the temporal relationship of data, in particular for PSA values. For example, high PSA value is an important data point but needs to be accurately integrated into the database based on the date of assay. An early (pre-surgical) assay date would indicate its role in triggering an initial diagnostic biopsy procedure, while a late assay date could indicate post-treatment recurrent disease. Again the solution employed by the Resource to address data context was largely a manual process of data collection and entry. However, this method did allow "integration" of more data elements than needed for the Resource, including non-CPCTR data elements. Yet for each site these additional data elements provided integration with other data sources and verified data integrity. Future solutions to these barriers include automated data retrieval through electronic queries of existing systems (i.e. EMR and the Enterprise Tumor Registry). These would require the use of messaging standards (i.e. HL7 and DICOM), and vocabulary standards (i.e. SNOMED and UMLS). While ideal, this process is slowed by the presence of legacy or unstructured systems using free text rather than structured data fields, which make searching for cases very difficult within these databases. In these situations manual annotation may still be required.

De-identification involves the separation of clinical (identified) data sets residing in the medical records from the research (de-identified) data sets that are available for the biomedical research community. This process is done with honest brokers who can collect clinical data (integration), identify the integrated data sets with a separate (research) numbers, and then "remove" the clinical identifiers before the integrated data is made available to the researchers. Multiple research numbers may be associated with the same set of integrated data, if necessary. For example, the CPCTR may share their complete de-identified data sets with another multi-center collaborative tissue bank, such as the Pennsylvania Cancer Alliance Bioinformatics Consortium (PCABC)[30,31]; the CPCTR gives a random 10 digit de-identification number for a particular case, which can then be given a second random system generated 8–10 digit number by the PCABC for their database. This process of de-identification is also preferred over anonymization (vide supra), because it allows for re-identification of cases by the honest broker for the collection and linking of longitudinal and outcomes data.

Standardization is a process of ensuring that all data elements are understood exactly the same, as well as collected and implemented in an uniform manner. This process allows one to "map" local clinical data (that has been integrated), to a set of "Common Data Elements" for a particular research collaboration. Note that the clinical data representation maybe different from the research representation and that there may be more than one research representation, for example:

▪ Clinical free text → Research CDE

▪ Clinical CDE → Research CDE

In CPCTR, many groups re-engineered the clinical system/workflow to make it consistent with CPCTR CDEs. But it is wise to allow some flexibility between the two because they are used for different purposes (and because there may be more than one research data set). The process of standardization was done by 1) redesigning clinical systems (for capturing synoptic pathology reports), 2) accepting clinical data elements into the central database (treatment and follow-up data from Tumor Registry) and 3) providing manual transformation of clinical data to the CPCTR paper forms.

Although, the process implemented by the Resource was successful for collecting data on prostate cases, it does not scale. While the design of common data elements and database construction were defined components that could be completed with only minor ongoing revisions, the subsequent data integration and annotation required extensive manual input. The amount of time needed to perform both data identification, integration, and input rose exponentially with the complexity of the patient population and their healthcare habits. Furthermore, as more new cases are entered, keeping up with activities related to ongoing annotation of the older cases while adding the newer cases becomes increasingly time and labor intensive. This is most true for data elements related to the patient's outcome, treatment and tumor markers data. Over time, an additional problem seen by the Resource was the inability to identify PSA values for some cases due to patient healthcare habits. Patients would leave the geographic areas or health systems after their initial treatment without providing follow-up information. This limited the amount of outcomes data that could be obtained from the patient's local medical records.

If this process were repeated, the Resource would advocate for time to perform medical records and lab information systems prescreening to determine if relevant clinical data could be found in existing clinical systems. If so, then the Resource would advocate for automated mechanisms to collect and integrate this data into a research database either at the intra- or inter-institutional level, with subsequent de-identification and transformation mapping to the CPCTR common data elements.

### Merging of local datasets to create the central dataset

The collection and merger of CDE data from multiple institutions revealed that variations in CDE interpretation can result in loss of CDE consistency and quality. While local annotation processes guarantee uniform local data collection this can still vary across institutions. There needs to be the collection and local storage of metadata for each of the CDEs and common QA protocol across institutions for the overall data collection process to make sure that each site interprets and reports the CDEs in the same manner. For example, at a single institution the Anatomic Pathology -Lab Information System and the local tumor registry may have data available for the same CDE regarding tumor histology or staging information. If contradictory data exists, the local data manager would need to know which metadata source would take priority and should be used to report the CDE to the Resource. The criterion applied for these decisions has to be standardized across the institutions and common to the entire Resource. Often this is an evolving process, as during initial data collection institution specific nuances in data storage and collection are identified, that may require specific decisions at the organization level. Verification of the accuracy of the final CDE data selection can only be accomplished through an analysis of the local metadata. Thus the requirement for metadata collection. Detailed discussion on the importance of metadata have been described elsewhere[13].

In lieu of strict guidelines for the implementation of a common data entry application created with MS Access, the Resource established standardized methods for data transmission to allow monthly updates to the central database. This allowed each center the flexibility of using different database technologies within their own organizations and permitted each institution to locally keep the honest broker's code book for linking the IMS de-identified case numbers to the patient identifiers. Thus, the de-identification process occurs locally at each site, and the data is not (and cannot be) identified centrally. In other words, the local data sets are independent and non-overlapping. One weakness of this system would be the potential of a patient to travel from one institution's healthcare system into another participating institution's healthcare system, thus becoming a duplicate case in the Resource. Only through an examination of the case CDEs for identity could such cases be identified. In the case of the CPCTR the geographical distribution made such cases negligible.

### Standardization and quality assurance of processes

To maximize the usefulness of biospecimens, especially for genomics and proteomics studies, it is crucial to collect extensive and accurate clinical annotation for all samples banked for research. The correlation between the research results and the annotations provided with tissue samples is highly dependent upon this process. The CPCTR differs from most other tissue resources in that (1) all accrued cases have undergone standardized pathology review, (2) the clinical data have been carefully collected using a standardized quality-controlled method, and (3) cases have been accrued across five states, from four academic centers, and from more than 15 hospitals. This wide range of cases eliminates the bias that may exist in resources populated by cases from single institutions. The implementation of QA protocols at the various levels discussed above, including automated scanning for logical errors in submitted data, selective review of case data, and independent review of randomly selected cases, provides a degree of confidence in the quality of the annotated specimen data. These processes also allowed the CPCTR to share data through sample query tools created for and available from the Resource.

It is also important to note that the CPCTR seeks to provide biospecimens for experimental studies that relate prostate cancer with associated clinical data. Since the resource provides clinical data with all tissue requests, investigators requesting only biospecimens without a need for clinical data were directed to alternative biorepositories, as shown in table 1. Since not all experimental tissue studies require extensive clinical annotations, provisions to address this issue need to be incorporated in future enhancements of the central database. For example, when a case is submitted to the central database, it can be marked as platinum, gold, silver, or bronze based on the quantity and quality of the associated clinical data. Platinum cases would represent those specimens with extensive associated clinical data. Such samples would be most appropriate for studies seeking correlation with detailed clinical outcomes parameters, either due to the large cost or nature of the experiment (genomics or proteomics studies), or based on previous study data that indicated biomarker importance related to a specific subset of clinical information (i.e. a relationship to PSA recurrence in high Gleason grade cancers). Bronze cases would represent specimens with the least amount of associated clinical data, and would be supplied for preliminary or exploratory studies. The gold and silver cases would represent specimens with intermediate levels of clinical data annotation. Having such a tiered system would allow biorepositories to tailor tissue distribution to investigators based on their specific research needs. Lastly, more work could be done to enhance data quality. Specific areas of improvement include the temporal collection of data related to both tissue acquisition along with patient disease history, and treatment changes, and outcomes. Such detailed temporal data will be necessary for accelerate the translation of research discoveries into clinical practice.

### Presentation of the central dataset

The collection of robust specimen data is of little value if the scientific community cannot access and examine the data during the course of experimental study design. Given the distributed nature of the CPCTR biorepositories, the use of a central database and web-based query tools that shared data with its user community was crucial to the success of the Resource. These query tools required specific features such as rapid performance, robust security features, and expansion capabilities for incorporating new data elements or integrating existing system features. Using the Oracle database platform for the query tools allowed the central database to develop layered web-based query tools to share data not only between the Resource members, but also with approved research investigators. The ability to define user access level to the data sets is essential for data monitoring and privacy issues. For example, the Resource allows public access to the database from its public website [27] using any of the approved 145 CDEs through its query tools, but the results are shown as basic summary statistics for a limited number of key data elements. The approved-investigator query tool is password protected and displays a more detailed query results for each case. Of note, interested investigators were encouraged to submit a full application describing their research protocol and tissue request without having reviewed the detailed case annotation, thus eliminating the possibility of case selection bias. Once cases were distributed to the approved investigator, they were provided web access to detailed datasets related to their set of cases.

All the data present in the Central Database represented de-identified HIPAA compliant data. Additional measures were also taken to transform the data sets centrally to meet HIPAA and IRB regulations. For example, all time periods were entered as plus or minus months from diagnosis. For example the "date of first recurrence" was not entered as a date but rather as "months from diagnosis" (i.e. Date of First Recurrence = 67 months). The "age of patient" for each case was displayed in ranges of decades (i.e. Age = 50–60 years). This data system structure and its protections were put in place to prevent "data-mining" for individual patient identification. Thus persons who had obtained privileged health care information regarding a specific patient would not be able to identify that patient using a single data element (such as a PSA value).

### Potential extensions of the dataset

Although the Resource has only recently initiated limited integration of other potential datasets that are associated with some of the specimens (or patient), the ability to extend current datasets easily has many advantages, including in its operations. Some of which include: 1) proper allocation of new specimens to investigators based on level of annotation; 2) Resource members and investigators can discuss the possibility of using the same sample (patient) for a similar study (i.e. gene microarray) or request an alternative sample; 3) meta-analysis of previously published works via a central tool; 4) and linkage to parallel databases for associated datasets or computation services.

Based on the utilization experience of the biorepository, multiple different datasets will be generated that may contain a given specimen. Thus a given specimen could be included in two investigator's approved datasets (one frozen tissue, one fixed tissue), and could also be included in a tissue microarray. In each experimental case differing clinical data may be needed. Some of which may be specifically collected for a given experimental study prospectively. Thus is will be important to have an informatics infrastructure that is flexible enough to easily expand and contain datasets that are currently being collected and produced using the specimens from the Resource. The CPCTR database was structured to effortlessly allow expansion of new data elements for such provisions. Current efforts include the integration of the CPCTR TMA XML data exchange file [14,15] and whole slide images for virtual microscopy related to the slides and paraffin blocks used for making the Resource TMAs, including the QA slides of various levels cut from the TMA blocks.

Although, the CPCTR requires all users of the biorepository to acknowledge the Resource in any publications that may result from Resource specimens, no requirement is made for data sharing by individual investigators. However, the database is structured to easily integrate references to published works or HTML links to raw data files generated for each originating specimen (and patient) used from the Resource. This would be important especially for linking high-throughput data sets such as gene microarray or proteomics datasets. Unlike classical tissue annotation and clinical data, these high-throughput techniques generate a large amount of well formatted data, typically for a relatively small number of tissue specimens. These data require significant computation analysis, usually using different approaches. Thus, this valuable information can be linked to the Resource's database through parallel databases that would allow keeping the high performance activities of the annotated tissue database, while sophisticated data analysis tools operate independently and provide links to other public databases [32]. Future steps that could be considered would include encouraging or requiring researchers to provide experimental datasets for re-annotation with specimen cases. While currently outside the scope of the Resource, such future data integrations would enhance the value of the specimens requested for subsequent studies.

### Management of marketing operations

Specifically, taking marketing surveys through informal calls to potential investigators that may use the Resource and accessing the needs of the research community with the members of the REP, allowed the Resource to re-direct much of the groups activity based on the needs of the user community. For example, the CPCTR was originally modeled as a complimentary paraffin tissue resource to the CBCTR [16,17], thus initially the Resource focused on archived cases that had a minimum of 5–10 years of follow up data. However, with new biotechnologies having emerged (i.e. gene microarrays), the need for banking frozen tissue was a direct result of meeting the impending research demands. Although, the Resource currently has over thousand cases with frozen material with 2–5 years of follow up information, the utilization of this material has been limited to date. This is partly due to the expensive costs involved with not only with the researcher (i.e. microarray chips), but also many of the logistics involved with collection, processing, and storage of the frozen material. Consequently, with additional informal surveys taken at large scientific research meeting, the Resource is currently focused on making several different tissue microarrays, including Gleason, ethnicity, outcomes, and hormone refractory TMAs.

Since the purpose of the CPCTR is to provide researchers with access to primary prostate cancer tissue and associated data, a major priority is to disseminate information about the availability of the Resource's specimens and data. Advancements in technology and the evolution of the Internet have provided a cost-effective means of disseminating information quickly and accurately in electronic formats using websites. By providing the web address for the CPCTR on flyers, bookmarks and other marketing items distributed at research meetings or via mass mailings, the Resource was able to reach other potential investigators who were informed by their colleagues of the CPCTR activities. The Resource was able to access the success of these activities by the number of inquires that persisted weeks after major marketing activities via emails or web visits to the Resource's homepage or public database.

By utilizing email tools, database reports, electronic surveys, as well as personal interactions at scientific conferences, the Resource gathered information from potential users of its tissues and their specific usage in research projects as well as from previous users of the quality of the services rendered. By adapting to the research communities' current needs as well as accessing their future needs was key to much of the Resource's success. This process allowed the Resource to serve the research community in an effective manner. The value of this process cannot be overemphasized.

## Conclusion

The CPCTR represents one of the largest sources of pathology-characterized archival prostate cancer tissue with associated follow-up data in the world and serves a broad base of researchers using a wide range of experimental methods (genomic, proteomic, histopathologic, validation and outcomes measures). The Resource has developed a highly effective informatics infrastructure that allows for efficient governance, standardized capture of data, and detailed standardized annotation of cases across multiple cooperating sites. This infrastructure includes an operations manual, a histopathology guide, and a database with common data elements for characterization of tissue samples and clinical follow-up data, and a quality assurance process. The uses of open database query interfaces that allow for user queries of Resource specimens while protecting patient confidentiality have been pioneered. The CPCTR has also initiated and developed of the use of internet-based, whole slide image review for quality assurance of the pathology characterization. These tools allow the CPCTR to function effectively across cooperating organizations and represent a "virtual" bank as set out in the original RFA. Furthermore, an online process for requesting samples and data by potential users has been developed and involves a research evaluation panel (REP) of five independent outside experts on prostate cancer, biostatistics, and pathology. Finally, a variety of tools have been developed to market the resource, including brochures, a website, and a booth that is being used to market the resource at scientific meetings. As such the Resource offers an important knowledgebase for the development of an integrated tissue banking program. Such knowledge is available for the benefit of other tissue banking efforts through the members of the Resource and their associated publications.

## Abbreviations

AJCC – American Joint Committee on Cancer

CAP – College of American Pathologist

CBCTR – Cooperative Breast Cancer Tissue Resource

CDE – Common data element

CHTN – Cooperative Human Tissue Network

CPCTR – Cooperative Prostate Cancer Tissue Resource

DB – Database

DCTD – Division of Cancer Treatment and Diagnosis

FCCC – Fox Chase Cancer Center

HIPAA – Health Insurance Portability and Accountability Act

HL7 – Health Level 7

HTML – HyperText Markup Language

IATA – International Air Transport Association

IMS – Information Management Services, Inc.

IRB – Institutional Review Board

LOINC – Logical Observation Identifiers Names and Codes

MD – Maryland

MS – Microsoft

NAACR – North American Association of Central Cancer Registries

NCI – National Cancer Institute

NCICB – National Cancer Institute Center for Bioinformatics

NIH – National Institute of Health

PCABC – Pennsylvania Cancer Alliance Bioinformatics Consortium

PL/SQL – Procedural Language/Structured Query Language

PSA – Prostate Specific Antigen

QA – Quality assurance

QC – Quality control

REP – Research Evaluation Panel

RFA – Request for Applications

SPOREs – Specialized Programs of Research Excellence

SNOMED – Systematized Nomenclature of Medicine

TJU – Thomas Jefferson University

TMA – Tissue microarray

TNM – Tumor/Node/Metastasis

UMLS – Unified Medical Language System

UPENN – University of Pennsylvania

UPITT – University of Pittsburgh

## Competing interests

The author(s) declare that they have no competing interests.

## Authors' contributions

AAP and JRG contributed equally to the first draft of this manuscript. MJB, who was the chair for the Coordinating Committee, was responsible for leading the efforts of developing the requirements for the central database. RG was responsible for overall design of the central database. MWD lead efforts for developing the PSA algorithm. AVP, RD, JJB, JM, AKB, and JO provided assistance in the developing the database requirements and incorporation of other existing standards. All authors reviewed and commented on successive drafts of the manuscript and have provided the first author with approval of the final manuscript.

## Pre-publication history

The pre-publication history for this paper can be accessed here:



## Supplementary Material

Additional File 1 **CPCTR Manual of Operations**. The Manual of Operations details the governance, protocols and guidelines that were created and used by the Resource. Click here for file

## Figures and Tables

**Table 1:** Tissue requests made to the CPCTR. A total of 131 tissue requests have been made to the CPCTR. The Resource has determined 77 of the total tissue requests to be eligible for which tissues are available for the research investigators. Additionally, there were 54 requests made that were determined to be ineligible by the Resource.

5	Full Applications (REP-approved and shipped to investigator)
21	TMA Requests (REP-approved and shipped to investigator)
19	Short-Form Requests (PI-approved and shipped to investigator)
2	Request approved (not shipped): Investigator to notify us when funds are available
2	Direct collaboration with a CPCTR institution
12	Letter of Intent (LOI) approved by REP, asked for Full application
16	Asked to Revise LOI or additional information requested by REP panel
**77**	***Eligible requests***

	
16	Referred to other resources (ie.CHTN)
7	Denied
6	Withdrawn
25	Closed (No response >12 months)

**54**	***Ineligible Requests***

	
**131**	***Total Requests received***

**Table 2:** Consultation of expertise. Partial list of organizations and groups that have consulted the Resource.

Pennsylvania Cancer Alliance Bioinformatics Consortium (Drexel, FCCC, PSU, TJU, UPENN, UPITT)
Australian Prostate Cancer Bio-Resource
The Georgia Cancer Coalition
The Kentucky Lung Cancer Research Program
The National Biospecimen Network (NBN) SPORE pilot project
Memorial Sloan-Kettering Cancer Center
H. Lee Moffitt Cancer Center esearch Institute
