# Supplementary material for: An informatics model for tissue banks – Lessons learned from the Cooperative Prostate Cancer Tissue Resource
Source: BMC Cancer. 2006 May 5;6:120. doi: 10.1186/1471-2407-6-120 (PMC1484486; doi:10.1186/1471-2407-6-120)
Supplement: Additional File 1 — CPCTR Manual of Operations. The Manual of Operations details the governance, protocols and guidelines that were created and used by the Resource. [file 1471-2407-6-120-S1.pdf]

National Cancer Institute
Cooperative Prostate Cancer Tissue Resource (CPCTR)

Manual of Operations

VERSION 11/04/03

TABLE OF CONTENTS

Page

1. CHARTER 1

    1.1 Resource

    1.2 Statement of Purpose

    1.3 CPCTR Membership

    1.4 Coordinating Committee

    1.5 Research Evaluation Panel

    1.6 Central Data Center (IMS)

    1.7 Meetings

    1.8 Publications

    1.9 Conflict of interest

2. Tissue Resource (Including tissue microarray) 6
3. Human Subjects 13
4. Administrative Tracking (Letters of intent and application) 20
5. Marketing (World-wide web) 29
6. Research Evaluation Panel (Conflict of interest clause. Review Guidelines) 32
7. Central Data Center & Common Data Elements 34
8. Histology Manual 40
9. Appendix (Letters)…………………………………………………… 51

NATIONAL CANCER INSTITUTE

COOPERATIVE PROSTATE CANCER TISSUE RESOURCE

SECTION 1. CHARTER

1.1 The National Cancer Institute Cooperative Prostate Cancer Tissue Resource is henceforth referred to as "Resource."

1.2 STATEMENT OF PURPOSE

The Resource is designed to provide large numbers of prostate cancer specimens with associated pathologic and clinical data to support well-designed studies that might not be possible with limited local resources. Such studies should improve the ability of clinicians to obtain an accurate biological classification of prostate cancers and improve treatment decisions. The Resource will support studies that involve: 1) developing and validating new diagnostic or prognostic markers or 2) determining the biologic role of diagnostic or prognostic markers. In addition, the Resource will welcome requests to support any promising prostate cancer research.

The Resource will implement quality control of both archival paraffin embedded specimens and data. It will carefully review cases to provide reliable descriptors of histology, extent of prostate involvement by cancer, high-grade dysplasia, tissue uninvolved by cancer, and metastatic disease. It will routinely update and check the accuracy of clinical, demographic, treatment, and outcome data. For prospective collections of paraffin embedded and frozen specimens, data will be collected and entered into the specimen database as it becomes available.

CPCTR procedures shall not compromise the tissue diagnosis or confidentiality of any patient and the CPCTR will maintain the integrity of all diagnostic specimens.

1.3 CPCTR MEMBERSHIP

A. Institutions:
Institutions comprising the Cooperative Prostate Cancer Tissue Resource (Resource) and funded under the request for cooperative agreement applications (CA-99-012) These are: George Washington University Medical Center, Washington, DC; Medical College of Wisconsin at Milwaukee, WI; New York University School of Medicine, New York, NY; University of Pittsburgh, Pittsburgh, PA.

B. Responsibilities of Awardees:
The Principal Investigators in cooperation with other members of the Coordinating Committee are responsible for developing the details of the Resource operating policies, including defining objectives and approaches, planning, implementation, marketing the Resource specimens, and establishing

quality assurance measures for all aspects of the operation of the Resource. The Principal Investigators must assure that designated Resource tissue remains available and that the relevant clinical data, as designated by the Coordinating Committee, is readily obtainable. Regular progress reports, at intervals designated by the Coordinating Committee, shall be submitted to the Coordinating Committee. Awardees retain custody and primary rights to data and specimens developed under these awards, subject to government (e.g., NCI, NIH, PHS) rights of access, consistent with current DHHS, PHS, and NIH policies.

1.4 COORDINATING COMMITTEE

1. The Coordinating Committee is comprised of two members from each of the four institutions, one of whom must be Principal Investigator, plus one National Cancer Institute representative, AKA, Program Coordinator. Additional members may be added to the committee by a majority vote of the existing committee members. The Program Coordinator is appointed by the Associate Director of the Cancer Diagnosis Program, Division of Cancer Treatment and Diagnosis. The committee will meet, in person, three times in the first year and at least twice a year in subsequent years with additional meetings as necessary. The committee shall hold conference calls as needed, usually monthly. The meetings are aimed at coordinating the activities of the participating sites, establishing new policies and priorities, and reviewing progress. At its initial meeting, and at subsequent anniversaries of that meeting, the Committee will elect a chairperson (who cannot be the Program Coordinator). The Chair is responsible for coordinating the Committee activities, for preparing meeting agendas, and for scheduling and chairing meetings.

2. The Coordinating Committee will act as the governing body of the Resource developing operating policies, which must be implemented by the Principal Investigators at each participating site. It will develop a Manual of Operations for establishing uniform procedures to accession, process, and distribute tissue, uniform quality control methods and rules for access to the clinical and outcome data associated with the accrued cases. The Coordinating Committee will determine priorities for applications final approval based on the recommendation of the REP. Coordinating Committee review of operating procedures of the participating sites is to insure that they are compatible with the overall goals and policies of the Resource, the NCI, and the NIH, to define specific quality control and tissue processing procedures, to establish procedures for effective communication and other network policies as needed.

3. The NCI Program Coordinator's role is to coordinate, assist, and facilitate, but not to direct the activities of the Resource. The Program Coordinator attends and participates as a voting member of the Coordinating Committee and will facilitate access to the NCI central database contractor to assist in implementing the central database for the Resource. The Program Coordinator acts as a resource for

information about NCI activities and advises on the acceptability of the Coordinating Committees policies to the NCI. The Program Coordinator may review the activities of awardees for compliance with operating policies developed by the Coordinating Committee and may recommend withholding support, suspension, or termination of an award for failure to comply with such policies.

4. Any disagreements that may arise on scientific/programmatic matters (within the scope of the award), between award recipients and the NCI may be brought to arbitration. An arbitration panel will be composed of three members: one elected by the coordinating committee (with the NCI Program Coordinator not voting) or by the individual awardee in the event of an individual disagreement, a second member selected by the NCI, and the third member selected by the two prior selected members. This special arbitration procedure in no way affects the awardee's right to appeal an adverse action that is otherwise appealable in accordance with the PHS regulations at 42 CFR Part 50, Subpart D and HHS regulation at 45 CFR Part 16.

1.5 RESEARCH EVALUATION PANEL (REP) (subject to approval by REP)

The Research Evaluation Committee (REP) is an independent (extramural) group of experts that will be constituted by the NCI in consultation with the Coordinating Committee. The REP works independently but collaboratively with the Coordinating Committee. The REP is responsible for reviewing requests from investigators for tissues and clinical data. The REP then provides advice about the scientific quality and priority of the request to the NCI and Coordinating Committee. An NCI representative, the Program Liaison, will be designated by the Associate Director of the Cancer Diagnosis Program. The Program Liaison cannot chair the REP, but is a member of the REP and participates in developing recommendations to the Coordinating Committee about scientific importance of proposed assays and the design of proposed studies. Two members of the Coordinating Committee serve as ad hoc, non-voting members of the REP. Once the REP is established, it, with input from the Coordinating Committee, will select replacements for members who have completed their terms. Service on the REP is generally for a period of four years. The REP chooses one of its members to be chairperson for a term of one to two years. Operating policies for the REP will be determined by the REP, consistent with operating policies developed by the Coordinating Committee. The REP members will attend at least one Coordinating Committee meeting each year to help coordinate REP activities with those of the Coordinating Committee.

1.6 THE CENTRAL DATA CENTER

Data from the four member organizations is merged into a central database designed and maintained by IMS, the Central Data Center. The primary and most important purpose of the central database is to allow retrieval of the information needed to determine the availability of appropriate specimens and data for proposed research on CPCTR resources. The database

contains information about the available tissue blocks and associated clinical and follow-up data.

The Data Center responsibilities as directed by the NCI are as follows:

- Development and maintenance of a central database that can be queried for specimen availability and selection.
- Tracking and responding to inquiries regarding the Resource.
- Processing of requests and coordination of letters of intent, applications and shipments of specimens.
- Preparation of invoices, collection of payments and distribution of payments for shipped specimens.
- Development and maintenance of systems to provide updated information to the CPCTR and the NCI.
- Report generation
- Development and maintenance of a database that the research community can query directly via the Internet.

The Coordinating Committee develops the Manual of Operations which contains information on CPCTR policies and procedures. IMS maintains and updates the Manual and ensures that all members of the CPCTR have a current copy of the document for reference. All decisions regarding the group operations are documented in the Manual. Any revisions to the manual are dated to ensure that all members are utilizing the most recent version.

1.7 MEETINGS

The Coordinating Committee will meet at least three times in the first year and twice a year subsequently. Additional meetings may be scheduled as needed by the Coordinating Committee or the NCI representative. Meetings may be held at any of the participating institutions or at another convenient location. These meetings are aimed at coordinating the activities of the participating institutions, establishing new policies and priorities, and reviewing progress.

The Chair of the Coordinating Committee will be responsible for coordinating the Committee activities, for preparing meeting agendas, and for scheduling and chairing meetings. An appointee is responsible for taking and distributing minutes of each meeting to all members. The NCI representative will attend and participate in all meetings of the Coordinating Committee and should be informed of major inter-group interactions. The Coordinating Committee will

prepare an annual progress report which will include individual reports from each participating site.

The regular meetings of the Coordinating Committee will be open to all interested parties from member institutions. The Committee can go into closed executive session of voting members at the discretion of the Chair or the NCI representative.

1.8 PUBLICATIONS

The role of the Resource should be acknowledged in all publications resulting from use of resource materials, but members of the Resource who make specific identifiable contributions may be acknowledged separately or included as co-authors.

Members of the CPCTR should be informed prior to any public presentation derived from group studies. The group should be acknowledged in every publication and presentation of data derived from group studies and individuals making significant contributions to the CPCTR studies should be appropriately recognized by co-authorship. Prior to initiation of studies, the participants will decide on anticipated co-authors. Final agreement will depend on appropriate participation in the protocol. The Coordinating Committee will resolve any problems arising among participants related to authorship of other aspects of joint studies.

1.9 CONFLICT OF INTEREST

The CPCTR participants agree to avoid conflicts of interest in the selection and execution of research projects. The NIH conflict of interest policy states that institutions receiving grant or contract support from the NIH will have a written conflict of interest statement and will notify designated institutional officials of potential conflicts of interest. There is no requirement that those conflicts be reported to the NIH if they can be resolved at the institutional level. The conflict of interest policy will follow the spirit of the NIH policy. Network members are expected to inform each other of any major commercial activities and when those involve the development of prostate cancer diagnostics, the type of assay or marker that is being developed.

Conflict of interest is defined as: 1. Any financial interest or arrangement with a company whose product is involved in or would be affected by a CPCTR study. 2. Any financial interest or arrangement with a competing company. 3. Any other financial connections, direct or indirect, or other situations that might raise the question of bias in the conduct or reporting of a study, including pertinent funding of a Network participant, a CPCTR institution, personal relationships, or direct academic competition. CPCTR participants are not required to reveal confidential or proprietary information, but are expected to indicate when they may be in conflict of interest.

SECTION 2. THE TISSUE RESOURCE

2.1 Overview

The CPCTR is a National Cancer Institute-sponsored tissue resource that combines tissue from prostate cancer specimens with associated cancer registry data to be used by interested researchers in investigational studies. The participating institutions expect to contribute cases with archival and frozen tissue to this resource. The resource development is initially directed toward obtaining archival tissue of cancer and control tissue from radical prostatectomy specimens. The later development of the resource will be directed to prospective banking of frozen prostate cancer tissue, derived also predominantly from radical prostatectomy specimens. Accrual of archival tissue from prostate biopsies, metastatic cancer tissue and normal tissue is also planned for the resource later in its development. The CPCTR functions as a virtual tissue bank in the sense that all specimens are kept at the four local institutions with an updated computerized database maintained centrally by IMS.

2.2 Pathology Subcommittee

The Pathology component of the CPCTR is directed by the Pathology Subcommittee, which acts as an advisory group to the Coordinating Committee (CC). The Subcommittee is comprised by a pathologist representative from each site, one of whom serves as a chairperson of the subcommittee. The chairperson of the Subcommittee is responsible for reporting progress and recommendations of the subcommittee to the CC, and for interacting with the chairs of other subcommittees on items of shared interest. The committee meets in person at on-site meetings and interacts frequently (on an ad hoc basis) via email or conference calls, with minutes recorded by the chairperson. The role of the Pathology Subcommittee is to develop the tissue-related operations of the resources – common data elements for pathology evaluation, pathology quality assurance systems, and methodology for tissue procurement, and tissue provision to investigators. The Pathology Subcommittee participants have collaborated to date to develop a common set of pathology database elements (as described below) for identifying appropriate tissues, categorizing them in a uniform fashion, and inventorying key paraffin blocks (designated “matrix blocks”) for rapid retrieval. In addition, the Subcommittee has introduced a system for quality assurance of pathology review.

2.3 Inclusion of Cases into the Resource

All prostate cancer patients are registered in the CPCTR if there is at least 5 years of clinical follow-up (except for frozen specimens), if they have at least one Matrix Block containing tumor, and if the critical Clinical Data Elements (CDEs) (see Section 7.7) are completed. In cases with only a single Matrix Block containing only a small amount of tumor, the specimen may not be available for distribution, owing to the need to preserve the diagnostic tissue in the Surgical Pathology files. The follow-up information required for the patient’s file would include vital status and PSA values for as many years as possible

2.4 Quality Assurance of Pathology Evaluation

At the time of entry, each site's pathologists review all surgical pathology reports, histological sections and paraffin blocks for each patient and completes the common data elements pertinent to pathology review. Each institution performs QA peer review per the individual institution's QA procedures.

The CPCTR has periodic QA assessments of inter-observer concordance for the Resource pathologists. These consist of 1) joint review of cases at meetings and 2) independent review of cases sent around from each site.

For joint QA review during meetings, Resource pathologists review 5 cases from each site, with emphasis on the 5 "matrix slides. Cases are selected to include areas of difficulty or likely diagnostic differences. Joint review of cases on a multiheaded microscope permits pathologist to discuss diagnostic differences and set thresholds. The cases are selected by pathologists at each site to include difficult cases and cases that illustrate areas of possible disagreement (with emphasis on assignment of Gleason grade, pathologic stage and margin status),

Independent review of cases will be performed at each site on cases sent from the other three sites at regular intervals (6 monthly). This will entail each site sending out the "matrix" and other pertinent slides of 5 cases to another of the resource sites, where pathologists formally review these cases. The IMS will randomly select the cases for QA review from those added to the Resource within certain cut-off dates. The reviewed material will consist of 2 to 5 matrix slides for each case. Record of review will be by completing the "matrix" fields and select "histology" fields of the CPCTR database. The completed data fields are then sent to IMS for analysis.

Any areas that show a high level of discrepancy will be determined by IMS and communicated to Resource pathologists. The pathology Subcommittee will then discuss their findings in the subsequent general meeting of the Coordinating Committee and provide a report with recommendations as indicated by their findings. This may entail a reassessment of diagnostic criteria and resetting of thresholds. Each site is responsible for correcting any errors discovered during the QA process and submitting their corrected database to IMS.

2.5 Pathology Guidelines Manual (PGM)

The Members of the Pathology Subcommittee will develop a set of guidelines during the first year of the project via a series of meetings, conference calls, and electronic mail exchanges. The illustrated CPCTR Pathology Guidelines Manual (PGM), which will be distributed to all CPCTR sites, will summarize the pathologic definitions necessary for completing the pathologic data and tissue inventory forms. All sites will use the same format for the pathologic data entry and record the same information. Each CPCTR pathologist will have a copy of the PGM and use it during the review of histological sections to encourage uniformity of classification at all

CPCTR sites. This manual will also be posted on the Internet for access by all participating pathologists and for those investigators who will utilize the resource.

The pathologic definitions and criteria are largely based on the Armed Forces Institute of Pathology (AFIP) Atlas of Tumor Pathology:Tumors of the Prostate Gland, Seminal Vesicles, Male Urethra, and Penis, Third Series, fascicle 28 (2000), by Robert H Young, John R Srigley, Mahul B Amin, Thomas M Ulbright and Antonio L Cubilla, a recently compiled reputable reference (provided by permission by authors). Individual pathologists of the resource have contributed illustrations for the manual. The manual serves to guide each CPCTR site pathologist by providing criteria and definitions for variables in the "matrix" and "histology" common data elements.

At each site, Resource pathologists review the surgical pathology report and all histological sections available - to accurately categorize each prostate cancer case, and to assign and characterize appropriate paraffin blocks, designated "matrix blocks", for the use of the Resource.

2.6 "Matrix" Paraffin Blocks (Paraffin Block Inventory)

The CPCTR pathologists have decided to "up-front" (at pathology review) to select paraffin blocks of interest for each case. This allows characterization of pathologic data for individual blocks and permits ease of retrieval of material. This system also allows standardization of inventory and retrieval of material because block information is maintained by the central database. The pathologist who reviews the case assigns two to five "matrix" blocks after review of all the histological slides in the case. The pathologist selects key slides according to whether they show specific features of the tumor (predetermined by the Pathology and Common Data Elements Subcommittees, as likely to represent features of interest to investigators) or high tumor volume. These slides, henceforth termed "matrix" slides, are used to retrieve the corresponding "matrix" blocks. The site coordinator is required to ensure that the specific blocks are in their block repository and contain sufficient remaining tissue. If certain blocks are not available or tissue has been depleted, then substitute slides and blocks on that case are utilized.

The number and selection criteria for "matrix" blocks differ according to the nature of the specimen – whether it be from a radical prostatectomy, needle biopsy, transurethral prostatectomy (TURP) or biopsy from a metastatic site (see supplements for selection criteria of blocks). One or two additional blocks are also selected for non-neoplastic prostate and one block of non-neoplastic lymph node tissues to enable provision of suitable control tissue to investigators.

Matrix Blocks are entered into the database according to their site-determined surgical pathology numbers and block designations. The Blocks designations are utilized solely for inventory purposes and are not made available to investigators to ensure adequate anonymization of cases.

2.7 Quality Assurance (QA) Procedures for Pathologic Data and Materials

CPCTR Principal Investigators regularly review Pathology data and registered tissues to assure the continued quality of the Resource. These audits, which occur during the semi-annual meetings of the CPCTR, consist of an examination of a randomly selected cases consisting of pathology slides and reports, and paraffin blocks.

Pathologic Review: The Pathology Common Data Element (Path CDE) Forms completed by the site's pathologists are reviewed by other pathologists, along with the slides and paraffin blocks. Any discrepancies are noted and discussed during the meeting, along with the QA analysis of the clinical materials conducted by the non-pathologist participants. The site's PI is responsible for correcting any problems in the specific case reviewed as well as re-reviewing cases if there is a systematic problem. Each CPCTR site has more than one pathologist entering cases and has developed a local QA mechanism for maintaining adherence to the Pathology Guidelines.

2.8 Provision of Material to Investigators

After the Research Evaluation Panel and the Coordinating Committee have approved an application for CPCTR specimens, the Central Data Center (IMS) randomly selects cases that meet the requirements as specified in the application. IMS circulates the case eligibility or selection criteria instructions to each CPCTR site so that specimen preparation may begin. After cases are selected for a supported study, the matrix blocks are retrieved and used to provide material. If there is insufficient tissue to prepare the requested number of sections, additional blocks may be selected to substitute for the depleted matrix blocks or the case may be re-categorized and another eligible case selected by IMS to take its place.

The cancer registry data is confirmed and updated at this stage. The review will, at a minimum, update the vital and cancer status of the case and check for logical inconsistencies or obvious errors in the initial submitted material. The requestor may also need further information than was available in the original data. If such information requires additional inquiries at each site, this information may only be provided through collaborative arrangements with individual sites.

The form in which tissues are prepared and distributed depends on the proposed methodology of the study for which the tissues are supplied. Whole tissue paraffin blocks are not distributed because of local regulations in member institutions of the Resource. The paraffin blocks however may be sectioned according to the requirements of investigators (thick or thin sections; on slides or in vials), or in certain instances sampled or dissected to create new blocks of tissue (such as tissue microarray blocks).

In order to conserve tissue particularly in the case of needle biopsy specimens, it is recommended that care be taken by histotechnicians to reduce loss of tissue at the microtome. Steps to decrease tissue loss such as adjusting the block orientation before cutting are advised.

2.9 Block/Section Designation of Tissue Samples sent to Investigators

Preservation of patient confidentiality mandates that material sent to investigators be stripped of original identifying numbers (surgical pathology number). Instead the IMS-assigned a 10 digit number for the case should be used on all sections or vials that are provided to investigators. Additionally, all sites differ in their block designation thus requiring a new and uniform designation to be assigned to each block or any sample derived from this block. This prevents an investigator determining from which site a sample has been sent and also diminishes confusion as to the nature of the sample.

Nomenclature to be used for blocks is as follows:

Radical prostatectomy specimens	
Matrix blocks of cancer:	M1 – M5
Benign Prostate tissue control:	B1, 2
Benign Lymph nodes:	BLN 1,2,3
Metastatic Lymph nodes:	MLN 1, 2, etc
Frozen prostate tissue, cancer:	FrM
Frozen prostate tissue, benign:	FrB
Frozen lymph node with cancer:	FrMLN
Frozen benign lymph node:	FrBLN
Frozen prostate tissue with HGPIN:	FrPIN

2.10 Procurement of Fresh Tissue from Radical Prostatectomy Specimens

The technique for procurement of tissue may differ slightly between sites, however all sites are guided by the same general principles for tissue procurement. These principles that each site ensures are:

a) Rapid procurement of tissue
b) Similar sampling techniques
c) Adequate characterization of frozen tissue
d) Adequate cold preservation
e) Appropriate measures for sending out specimens in cryopreserved state
f) QA procedures to ensure viability of tissue components

2.11 Rapid Procurement of Tissue

Pathologists at each site have investigated the steps and time duration for the transportation of a radical prostatectomy specimen to the pathologist - from the time it is removed from the patient until the time the pathologist sections it. Any measures to expedite this process have been implemented. The routine delivery of tissue to the laboratory may not be appropriate for these cases – and therefore different methods of transportation may need to be used. A record of the time lapse, from surgical removal until cryopreservation is to be performed for each case. Current studies focusing on RNA viability have noted isolation of intact RNA from tissues up to

1 hour (MCW) to 6 hours (Pittsburgh) after operating room procurement. While the former time period is favored, all tissues should be banked and time to harvest recorded, with subsequent stratification according to research needs determined at the time of allocation.

2.11 Sectioning Techniques

A standard protocol is followed at all sites for grossing the prostate gland. First the prostate gland is weighed, measured in three dimensions, and then its surface inked to allow evaluation of margins. The apex and bladder neck margins are then submitted for permanent sections, following which consecutive transverse sections are obtained of the remainder of the gland. At some of the sites (Pittsburgh, NYU), the capsule is first removed from the prostate gland prior to sectioning transversely. Sectioning is performed rapidly to enhance viability of tissue. The transverse sections of the gland are then inspected to allow appropriate tissue procurement.

2.12 Sampling Techniques

Members of the CPCTR have considered and compared two methods of procurement, namely sampling for “pure tumor” by macro-dissection at the time of procurement with routine sampling of alternate whole slices (without any dissection for tumor). Routine sampling of alternate whole slices has been selected by members of the CPCTR (i.e. to rapidly freeze and store non-dissected tissue rather than dissected tumor tissue), for the following reasons: 1) Alternate whole slices ensure a larger amount of tissue to be banked 2) Removal of a dissection step allows for a shorter time for procurement 3) A routine method of sampling eliminates the need for a pathologist’s input at the time of procurement and allows this step to be performed by a technician 4) The routine method avoids the need to separately procure control tissue. The recognized disadvantages are that 1) each site will not have an up-front knowledge of the exact amount of tumor tissue/ case 2) macro-dissection will have to be performed at a later stage 3) this method increases the amount of tissue stored and hence utilizes greater amount of space in liquid nitrogen storage freezers. Still the advantages of a routine method outweigh the disadvantages particularly at sites where procurement is performed at many different hospitals.

The protocol therefore is to freeze alternate tissue slices without pre-dissection for tumor. One or more slices of approximately 2 x 2 x 0.5 cm are stored.

Note that the procurement of tissue is not performed if there is any anticipation that it may compromise the patient’s diagnosis. Frozen tissue always remains accessible to the primary pathologist for retrieval for routine processing and analysis. This is particularly relevant to cases where there is no cancer in the permanent sections or there is a discrepancy between the biopsy and the radical prostatectomy findings that could be due to inadequate sampling in permanent sections.

2.13 Characterization of frozen tissue

The frozen tissue is characterized for the presence and extent of tumor by analyzing frozen sections of the corresponding tissue. This enables pathologists to deliver verified tissues for

research. Pathologic information is derived from the corresponding formalin-fixed processed tissue slides in order to complete "frozen tissue CDEs" which document pathologic details of likely use by researchers, and permit the resource to maintain an inventory of tumor tissue. It is understood that there is a certain level of error in the frozen tissue CDEs and the frozen section evaluation prior to disbursement of the tissues. The CPCTR has chosen this approach to reduce the time to tissue freezing and in the absence of prior knowledge of the actual needs of the requesting scientists.

2.14 Methods for cold preservation

Flash freezing of tissue is performed. Tissue is stored at –150c – in liquid nitrogen freezers, in vapor phase. Details of tissue storage containers and wrapping are worked out at each individual site so that the following guidelines are met: 1) stability without rupture 2) immunity from cross-contamination, and 3) compliance with human specimen handling as dictated by the local tissues committees.

2.15 Sending out frozen tissue to investigators

A. **Protocol for packaging of frozen tissue: as per the methodology stated in the *IATA Dangerous Goods Regulations: 41st Edition Effective January 2000***

Sending Biological Specimens that are NOT "infectious"

Biological Specimens that are not infectious are sent under "**Diagnostic Specimens**" regulations PI 650. (Reference 3.6.2.1.4)

UPS is **NOT** an IATA member and will not accept formalin or infectious substances; FED EX **IS** a member and will accept diagnostic specimens, infectious substances, and formalin. (Reference 2.9.4.5X-04)

Diagnostic specimens must be packed in combo packaging (inner and outer packaging).

A. **Inner packaging** comprising:

- **A watertight primary receptacle:** (i.e. plastic bag: The weld-seams and closures of such bags must be sift proof. Plastic bags must have a minimum thickness of 0.1 mm: Reference 6.1.5)
- **A watertight secondary packaging:** (i.e.: styrofoam container)
- **An absorbant material**-must be placed between the primary receptacle and secondary packaging. No absorbant material is required when shipping solid substances.

B. **An outer packaging** of adequate strength for its capacity, weight and intended use. (i.e. a certified UN fiberboard box)

When shipping with **dry ice,** the secondary packaging (styrofoam box) must permit the release of carbon-dioxide gas and therefore **SHOULD NOT** be taped shut. If **wet ice** is used the packaging must be leak proof. The primary receptacle must maintain its containment integrity at the temperature of the refrigerant as well as at the temperatures and pressure of air transport to which the receptacle could be subjected if refrigeration were to be lost. (Reference Packing Instruction 650)

Dry Ice **MUST NOT** exceed 5 lbs (Reference List of Dangerous Goods 1845) and the net weight **MUST** be marked on the outside of the box (Reference Packing Instruction 904)

Substances shipped at ambient temperatures or higher (i.e. cells): Primary receptacles include those made of glass, metal or plastic. Positive means of ensuring a leak-proof seal, such as heat seal, skirted stopper or metal crimp seal **MUST** be provided. If screw caps are used these **MUST** be reinforced with adhesive tape. (Reference Packing Instruction 650)

An itemized list of contents **MUST** be enclosed between the secondary packaging (styrofoam container) and the outer packaging (fiberboard box) (Reference Packing Instruction 650)

All labels and markings **MUST** be on the same side of the box (Reference 7.2.6.1.e)

Labels **MUST NOT** be folded or affixed that they are touching another side of the box (Reference 7.2.6.1.c)

Labels are **NOT** permitted to cover any writing or instructions on the box (Reference 7.2.6.1.a)

Labels **MUST** be a minimum of 100 x 100mm (**4 x 4 in**) (Reference 7.2.2.3)

A package containing "diagnostic specimens" **MUST** have the label **"DIAGNOSTIC SPECIMENS PACKED IN COMPLIANCE WITH IATA PACKING INSTRUCTION 650"** (Reference 7.1.5.1.g)

The full NAME AND ADDRESS of the shipper and cosignee **MUST** be written on the **BOX**. (Reference 7.1.5.1.b)

NOTE: Infectious tissues (i.e. Hep C, HIV) have a completely different set of regulations. These regulations are very extensive and will be reviewed for certification purposes and an outline will be handed out at a later date.

2.16 Freezer safety measures and Precautions in event of Freezer breakdown/ power loss

All freezers used should have a backup system and electronic/telephone notification system with at least two personnel contacts. While it is realized that not all disasters can be

avoided, in the event of a catastrophic breakdown sample recovery may be possible. Review of the temperature record to identify the warmest temperature achieved along with random sample viability studies for nucleic acid and protein stability should be undertaken before any tissues are discarded. Final decisions regarding tissue suitability for the CPCTR will be decided by the coordinating committee.

2.17 QA Procedures for stability of stored frozen tissue

Quality Assurance studies will be performed to measure the amount of mRNA in stored frozen tissue, based on the perceived needs of the research community and the instability of this nucleic acid. Analysis of total and mRNA (for example Ubiquitin mRNA) is to be performed on additional tissue (excess transitional zone prostate tissue) which will be banked on every 10th case, or in cases where there has been an excessive delay (greater than 1 hour) in procurement.

Another QA step may be required on tissue after it reaches outside investigators to determine decay of mRNA during transport. The “excess tissue” samples may also be used for this purpose.

One of the suggested protocols could use the following methodology.

Total RNA is extracted from these samples using standard protocols (the TRIzol Reagent method, Gibco BRL). Single stranded cDNA is synthesized with polyA oligonucleotide. RT-PCR is performed for Ubiquitin for 35 cycles under PCR conditions. The PCR conditions are 94°C for 1 minute, 5 cycles of 94°C for 30 seconds and 72°C for 4 minutes, 5 cycles of 94°C for 30 seconds & 70°C for 4 minutes, 25 cycles of 94°C 30 seconds & 68°C for 4 minutes. An ethidium bromide stained 1.5% agarose gel is used to separate and size the PCR products. The results of this experiment show good preservation of RNA in the banked specimens.

2.18 Tissue microarrays

On a regular basis, CPCTR sites will create tissue microarrays (TMA) for use by investigators requesting such specimens. These TMA include, e.g. cancers from random radical prostatectomies excised from patients with long clinical follow-ups, cancers from patients with clinical recurrence, and cancers from Caucasian and African American individuals.

A method is being perfected that will allow the use of needle biopsies to make TMA.

Cost for tissue microarray (TMA) slides and clinical information

$600 for a set of four slides for researcher, with annotation

$1,800 for a set of four slides for commercial enterprises, with annotation

3 free optimization TMA slides for testing probe, to all scientists, without annotation

$25 for additional optimization TMA slides for academic institution

$75 for additional optimization TMA slides for commercial enterprises

The first TMA announcement to the research community

Subject: Prostate Cancer Tissue Micro-Arrays: Newly available resource for your research

CPCTR Prostate Cancer Tissue Microarray (TMA)

The **National Cancer Institute** funds the **Cooperative Prostate Cancer Tissue Resource (CPCTR)** [www.prostatetissues.org] to provide prostate cancer tissue samples to researchers.

The CPCTR is now pleased to announce the availability of slides from **prostate cancer TMAs with associated clinical data.**

All researchers may apply for TMA slides (academic or commercial, US or foreign).

The TMA slide set contains

- Cancer tissue from radical prostatectomy specimens of **299 patients**
- Control non-neoplastic tissue from **benign prostatic hyperplasia (BPH)**
- Control non-diseased tissue from **organ donor** prostates
- Cores from two prostate cancer cell lines**: LNCAP and PC3**

The information provided for each case on the array includes over 20 data elements including: age at diagnosis, race, PSA at diagnosis, tumor size, TNM stage, Gleason score and grade, and vital status.

Further information, including costs, can be found at [http://cpctr.cancer.gov/FAQ.html#cost].

An application form for TMAs is available at [http://cpctr.cancer.gov/loi.html].

Please e-mail us at **ASK-CPCTR-L@LIST.NIH.GOV** if you have any questions about our TMAs or our large repository of paraffin-embedded and fresh-frozen prostate cancer tissue samples.

TMA Slide Storage and Shipping Protocol

TMA slides prepared at the Medical College of Wisconsin and are shipped by Dr. Milton Datta, Department of Pathology, Medical College of Wisconsin, 9200 West Wisconsin Ave, Milwaukee, WI, 53226

After a TMA block is prepared an initial run of 21 slides are cut and placed on plus charged (+) slides and used for initial quality assurance (see slide checking protocol). Upon completion of the initial quality assurance, the remaining block is step sectioned and the slices placed on sequentially numbered plus glass slides and stored as detailed below. Additional quality assurance checks are carried out at this time. Any additional sections or portions of sections produced during the preparation or cutting of the block are placed on plus (+) slides and stored for use in antibody testing.

Each positively charged (+) microscopic glass slide holds a single TMA paraffin section. Up to 100 slides are stored in a green polyethylene microslide plastic box (Fisher scientific cat # 03-448-5) that shield the slides from light and cushion them from breakage during shipping. Smaller boxes are utilized when only 10 or 20 slides are shipped. Each box is stored in a vacuum dessicator at 4C in the dark.

When a request for TMA slides is approved, MCW CPCTR TMA Coordinator opens the dessicator and box, removes the approved number of slides and ships them in a slide box placed in a Ziplock bag, packed and cooled with ice packs and shipped within a Styrofoam container by overnight express mail. This procedure is also described in the CPCTR MOO. After removing the slides selected the Coordinator closes the box containing the remaining slides and places the box back in the vacuum dessicator at 4C in the dark.

SECTION 3 HUMAN SUBJECTS

The CPCTR participants will ensure that the integrity of all diagnostic specimens is maintained and that no CPCTR procedure will compromise the tissue diagnosis of any patient.

3.1. REGULATIONS AND INFORMED CONSENT

The specimens and information collected by the CPCTR will be used for human subjects research and applicable laws and ethical requirements must be observed. While all specimens and clinical data are coded, a link to the participant/subject is maintained and CPCTR materials and information must be approved by IRB review. The common rule, 45CFR 46, the Federal Regulation for protection of human subjects in federally funded research does allow an Institutional Review Board (IRB) to waive the requirement for informed consent, as follows:

An IRB may approve a consent procedure which does not include, or which alters, some or all of the elements of informed consent set forth in this section, or waive the requirements to obtain informed consent provided the IRB finds and documents that:

- The research involves no more than minimal risk to the subjects;
- And the waiver or alteration will not adversely affect the rights and welfare of the subjects;
- And the research could not practicably be carried out without the waiver or alteration.

Recognizing that the intent of the IRB review process is to protect patients from harm due to medical research, CPCTR sites shall obtain appropriate IRB approval for all activities. The participants recognize that informed consent should be required whenever patient samples are collected prospectively, since patient care may be affected. For retrospective collection of residual diagnostic tissues, waiver of informed consent may be appropriate since patients are not harmed by the use of residual tissues for minimal risk studies. Some reasons to waive informed consent are:

1. Tissue banking with de-identification of tissues as used in the CPCTR poses no risk of loss of confidentiality of the subjects involved because:
   a. Confidentiality is adequately preserved; therefore
   b. Researchers will never have access to patient identification.

2. The waiver will not harm subjects, and will not impair medical care because:
   a. Diagnosis on the tissue has already been rendered, and the paraffin block is left over tissue that is often discarded after a period of time. Current College of Pathologists guidelines used by most laboratories in this country are to keep paraffin blocks for a period of at least 5 years;
   b. Familial studies cannot be done with this type of material since only index cases are available.

3. The research could not be carried out without the waiver without losing a unique and very powerful feature of this tissue bank, an unbiased collection of consecutive cases:
   a. Since most of the pathology specimens were accessed several years ago, it will be impracticable to contact all the patients or the families for retrospective consent;
   b. Bias would be introduced into the collection of specimens if only consented specimens are accessed into the tissue bank.

Some IRBs hold the view that calling family members to obtain consent may cause more harm than good. Patients and families may be upset by the information that their tissues are still in storage. However, waiver of consent is the purview of local IRBs.

Since an honest broker model is used at NYU to protect patient privacy, a mechanism exits to notify a patient or family if information of importance were obtained.

3.2 SPECIMEN COLLECTION

Only tissue in excess of that necessary for diagnosis will be banked by the CPCTR. The CPCTR will never accept tissues needed for patient diagnosis.

1. Retrospective Specimens

Retrospectively collected clinical specimens are paraffin blocks containing specimens from radical prostatectomy and transurethral prostatectomy (TURP). Paraffin blocks and unstained paraffin sections of radical cystoprostatectomy specimens, TURPs, prostate needle biopsies, and other biopsies or tissue specimens of metastatic prostate cancer sites may also be collected.

2. Prospective Specimens

Collections of radical prostatectomy specimens will begin in year one, frozen specimens, needle biopsies, and TURP specimens will be collected prospectively beginning in year two. In addition, prostate tissue from rapid so-called warm autopsies of prostate cancer patients and non-prostate cancer patients as well as from organ/tissue donors may be collected at some sites.

3.3 . CONSENT

The CPCTR sites will each determine the most expeditious and compassionate manner to request consent from patients at their participating institutions.

1. Retrospective Cases

When consent is waived by the local IRB, CPCTR sites may bank existing specimens without patient consent. However, if a patient must be contacted, in order to obtain

additional clinical information, each site will do so according to a written plan approved by their Institutional Review Board.

2. Prospective Cases

As noted above, written informed consent is required for all prospective collection of specimens and patient data. The methods by which informed consent for prospective cases is obtained will vary from site-to-site, depending on the requirements of local IRBs, local logistics requirements and other local conditions. In most cases, the most feasible approach is for the same people (e.g., urologist or nurse) to obtain informed consent at the time of the surgical consent. This approach also provides an opportunity to explain the differences between the two consents. The participating Institutions should assure that their consent process provides adequate time and information to allow patients to fully consider all of the issues involved with their participation.

3.4 PROTECTION OF PATIENT IDENTITY

Protection of patient identity is part of the consent process. Each institution has included consent language describing their procedures for protection of patient identity. An example, from one member institution, is as follows: "Your medical and research records may be provided to the sponsor, the United States Department of Health and Human Services and the Committee on Human Research of the NIH. The NIH may also review your medical and research records. Except for these entities, medical and research study records will be kept confidential unless you authorize their release or the records are required by law (i.e., court subpoena). You will not be identified (e.g., name, social security number) in any reports or publications of this study".

1. METHODS USED AT SITES

Patient specimens and data transferred from consortia sites to their lead institution will be identified only by accession number. Any information or identifier that could identify the donor, is retained by the donating site which has received the consent for the patient to participate in the CPCTR. No surgical pathology numbers, medical record numbers, etc, will accompany a case. Some sites will utilize some form of "The Honest Broker Method" to ensure the protection of patient identity. One version of this model is described in the Appendix to this section.

2. METHODS UTILIZED IN THE NATIONAL DATABASE

Patient identity is further protected since each case in the national database is identified by a code number. The only link is retained at the local member institution. The CPCTR data entry program, designed by Information Management Systems (IMS), assigns a random, ten-digit number as each case is entered by an institution. These numbers provide the only identifier for individual case data stored in the central database.

3. DISTRIBUTION OF SPECIMENS

Once the CPCTR Coordinating Committee approves investigators' submitted proposals, IMS will determine which CPCTR member(s) should be the distribution site(s) for the specimens and data requested. The institution holding the cases will be notified to disburse to the investigator.

The IMS random number identifier will be the only link between specimens and clinical data supplied to an investigator. The investigator will know the identity of the institution shipping the specimens from the return addresses.

3.5 PATHOLOGY REVIEW

1. INSTITUTIONAL REVIEW

Each participating institution will review all of their cases considered for inclusion in the CPCTR. The respective pathologist, at the participating site, will perform a detailed evaluation of all the slides submitted for each case. They will then select appropriate slides and blocks to be designated as "the matrix blocks" (representative blocks). These blocks will provide tissue types and materials considered essential by the group. These tissues are anticipated to help address some of the most common requests from investigators. This is addressed in more detail in the Pathology Section.

2. CENTRAL REVIEW

The Coordinating Committee of the Cooperative Prostate Cancer Tissue Resource, in conjunction with the Pathology Subcommittee of the Prostate Cancer Tissue Resource, will supervise the central review of cases. The currently envisaged protocol for central review includes 2 major mechanisms:

a. Review of cases selected by the Information Management Services (IMS). IMS will select cases at random, from all the cases submitted to be a part of the repository, for purpose of quality assurance and quality control. Such cases will be identified by CPCTR # only.

b. Consultation among institutions: Collaborating institutions will exchange cases for consultation and review. This is envisaged as a good mechanism to promote cooperation and interaction among participating institutions. It should also help to maintain uniform criteria and standards of histologic evaluation and assessment of the cases contemplated for inclusion in the prostate resource. Such cases will be identified only by CPCTR #.

Discrepancies in Review: The pathologic review of retrospective cases for entry into the CPCTR may identify a few cases in which the CPCTR result differs from the original

pathology report. In most cases, these changes are expected to be of no consequence to patient care. If a discrepancy has the potential to affect patient care and outcome is found, a letter will be immediately sent to the pathologist of record (original sign out pathologist) reporting the discrepancy. The case will not be included in the CPCTR, since the blocks may be needed for further evaluation of the case by the pathologist who first saw the case. The letter will be written by the CPCTR pathologist who reviewed the case and a copy of this letter maintained on file. Similar policies are used by other multi-institutional national protocols such as those of the Eastern Cooperative Oncology Group and the National Surgical Adjuvant Breast and Bowel Project.

3.6 . HUMAN SUBJECTS WORKFLOW SUMMARY

Maintaining patient confidentiality is a high priority for all CPCTR sites. Plans for handling specimens and collecting and transmitting information were developed with this in mind. The only linkage to patient identity is retained locally at the NCI funded institution (local site) in accordance with their IRB approved protocol. The NCI contractor, Information Management Services (IMS), maintains the central database for the CPCTR. Data submitted to IMS by the local sites consists of clinical, pathological, therapeutic, and demographic information, identified only by a randomly assigned ten-digit CPCTR case number.

The honest broker model requires that a group or individual other than the investigator protects the link between specimens and associated data and patient identity. In this way, knowledge of patient identity is kept from the researcher and any others without need to access that information, but the ability to gather additional follow-up information is retained. All local sites protect patient identity and pertinent staff have signed a confidentiality agreement stipulating that they will not reveal patient identity. In addition, some participating institutions have instituted more stringent "honest broker" arrangements to further protect patient identity.

Under no circumstances will the investigator be given any information that could allow them to establish the identity of patients. Once the Research Evaluation Panel approves an application to receive CPCTR specimens, IMS will identify appropriate cases search in the central database. IMS will then provide each participating site with a list of their cases, identified only by CPCTR number. The participating sites will then prepare the requested specimens to meet investigator requirements and ship them. IMS will send the patient data to the investigator. Specimens and data are identified to the investigator only by the random CPCTR number.

3.7 APPENDIX: "THE HONEST BROKER CONCEPT"

CPCTR sites ensure protection of patient identity through "The Honest Broker Concept." In order to protect patient identity for specimens and data in a tissue bank, the following prerequisites must be met:

1. Patient confidentiality must be protected
2. Investigators must have access to future clinical outcome.

The combination of items (1) and (2) seems to be impossible since they are mutually exclusive. Availability of confidential information sounds like an oxymoron. It is possible however to conciliate these two needs when one uses the honest broker or tissue bank trustee to provide a barrier between patient confidential information and researchers. The honest broker can be an individual or a group. In most cases provisions are in place for the local CPCTR grantee site to act as the honest broker. By using the honest broker to ensure one way flow of information, confidential information and patient identity can be protected.

In one institution, this concept is made even more stringent by having only one tissue bank trustee. One of the tumor registrars is designated as the tissue bank trustee since tumor registrars, by the nature of their job and by federal mandate already have access to clinical information on cancer patients and do not have access to the results of research data for tissue bank samples. The trustee is the only person who can link a patient with the number that identifies that patient. The trustee system ensures that new clinical outcome information can be added to a file identified only by a code number, not a name. In the extremely rare event that important research data becomes available and it becomes necessary to inform the patient or his survivors, a fail-safe mechanism remains for that information to reach the interested party.

The purpose of the honest broker or tissue bank trustee concept is to protect patient confidentiality while still providing a mechanism to access clinical information.

SECTION 4. ADMINISTRATIVE TRACKING

4.1 Overview

IMS tracks, responds to, processes and coordinates the receipt, review, and processing of requests for Resource specimens. Several tasks are involved in this coordination.

4.2 LOIs/Application Requests

IMS receives and responds to inquiries about the CPCTR and requests for obtaining specimens. IMS tracks all requests and responses to ensure that inquiries are processed efficiently and responded to in a timely manner. Inquiries are received via telephone, letter (rarely), and (most often) electronic mail through the CPCTR web site. IMS responds to inquiries received directly or, if necessary, forwards them to the NCI or the Coordinating Committee for advice on the appropriate action to take. IMS responds to the inquiring investigator by either directing him/her to another resource (if the CPCTR is not the appropriate one) or by providing information on how to submit a Letter of Intent (LOI).

LOIs are required before a researcher is encouraged to submit a complete application. LOIs are numbered sequentially (L-nnnn) and forwarded upon receipt to the NCI Research Evaluation Panel (REP) coordinator for review. IMS sends a response to the investigator acknowledging receipt of the LOI. If the NCI determines that the CPCTR is the appropriate resource for the request, then the LOI is distributed to all REP members for review. The composition and role of the REP and the review process are described in Section 4.3.

IMS notifies the applicant once a recommendation has been made regarding the LOI. If the LOI receives a favorable review, the NCI REP Coordinator assigns a REP member as guide to work with the applicant and provide feedback to the applicant on the issues raised by the REP. IMS then sends the applicant (with a copy to the NCI and the designated guide) the necessary information and forms needed to submit a full application. If the LOI does not receive a positive review, the LOI is forwarded to the Coordinating Committee for final discussion and consideration.

Upon receipt of completed signed applications, IMS performs an initial quick review for completeness. IMS returns incomplete applications to the investigator for completion. If a completed application is received, it is then processed for review. IMS notifies the investigator regarding the receipt of his/her application and the date of review. The application is then numbered according to the LOI number but without the preceding “L” (nnnn).

IMS performs an initial search of the central database to determine the number of available specimens that match the criteria specified by the applicant. IMS reviews the application for completeness, page numbers the request, and forwards it to the NCI REP Coordinator. Following NCI review, the application is distributed to all members of the REP, to the Coordinating Committee Liaisons and the NCI Coordinating Committee Representative. IMS participates in the review of the application and then sends the application to the remaining CC members prior to their discussion of the REPs recommendation. When the final review of the application is completed, IMS formats the

letter to the Investigator from the NCI regarding the status of his/her review. The status of LOIs and applications is summarized and distributed regularly to the Coordinating Committee.

4.3 Review Procedures and the REP

As described in the Section 1, the Research Evaluation Panel (REP) is charged with reviewing the scientific merit of requests for CPCTR specimens and making recommendations to the CPCTR Coordinating Committee (CC) regarding whether the requested specimens should be provided. The REP is composed of extramural scientists with varied expertise including laboratory science, prostate pathology, prostate oncology and statistics. One member of the REP must be an extramural program staff member of the NCI. Service on the REP is generally for a period of 4 years. One member of the REP is chosen by the REP to be chairperson for a term of one to two years. The NCI extramural program staff member may not serve as chair and does not rotate off the panel. Replacements for members who have completed their terms are chosen by the NCI based on recommendations from the REP and the CC.

The members of the REP follow established review guidelines and there is a conflict of interest agreement to which all members adhere. The review process is as follows:

1. Letters of intent are submitted to the support contractor. The LOI must contain the following information: the approximate number and types of cases required; rationale for the number of cases; an estimate of the number of sections required; an outline of the aims of the proposed research; a brief description of the technical approach; statistical design, and a statement that the technique(s) can be applied to paraffin-embedded specimens. If the LOI contains the necessary information and the contractor determines that the types of specimens and data requested can be supplied by the CPCTR, IMS sends the LOI to the designated NCI REP Coordinator (Barbara Conley) who checks if everything is in order and whether there are conflict of interest issues with members of the REP.
2. The NCI REP Coordinator forwards the LOI to the REP with a deadline for comments and recommendations as to whether a full application should be invited (generally within 2-3 weeks). If there are serious questions, a conference call is arranged.
3. The Chair and/or the NCI REP Coordinator (Barbara Conley) assign a member of the REP as a guide to assist the applicant in preparing an appropriate application, including discussing questions raised by the REP members during their review of the LOI. A statistician is assigned to the request.
4. If the REP determines that a full application should be submitted, the contractor informs the applicant and provides the application forms. The investigator must have a guarantee of funding before an application will be considered.
5. When the application is received, the contractor sends it to the NCI REP Coordinator (Barbara Conley) for review of completeness and eligibility.
6. The NCI REP Coordinator (Barbara Conley) discusses the choice of primary and secondary reviewers and timing for review with the REP chair. One reviewer will usually be a statistician. The necessary information is conveyed to IMS who sends the

application and cover letter with review assignments by express mail to the members of the REP.

7. Review of the application is generally by phone conference call, scheduled generally within 4 weeks of the time the application is mailed out. If the application is received at a time when the REP is to meet jointly with the CC, then the REP will meet separately to conduct the review.
8. The REP reviews the importance of the basic questions being addressed by the study and identifies study design problems and areas that need improvement. The REP makes a recommendation to the CPCTR CC that the applicant should receive the requested specimens and data, that specimens should be provided only if the study design problems are addressed, or that specimens should not be provided. The CC takes into account whether there is a judicious use of the specimens.
9. The REP meets one time annually with the CC to discuss REP procedures and other issues related to the CPCTR.

4.4 Specimen Selection and Preparation

Once an application has been approved by Coordinating Committee, a list of specimen selection criteria and exclusions is prepared by the NCI statistician and IMS. The list of criteria is distributed to the NCI officers, and the investigator-applicant for verification. The investigator is asked to confirm the specific clinical and pathological data elements required. The investigator is also asked to provide details on the preparation, storage, plus preferred method and timing of specimen shipments. This information will be communicated to the CPCTR sites by IMS when case review has been completed. IMS will supply an estimated cost to the investigator for slide preparation and shipping costs. A purchase order number or sufficient proof of payment is required prior to the sectioning of any blocks.

Upon verification of all selection criteria and proof of payment, IMS randomly selects the required number of eligible cases plus an additional 10% to be used as possible replacement cases, if necessary. IMS provides each CPCTR member with the list of cases and replacements selected from their site along with instructions for verifying eligibility requirements and logging the specimens shipped.

A data review form similar to the audit form is generated for each of the cases selected. The report lists the data variables requested by the applicant and is to be completed at the time of case review. All eligibility criteria is verified and appropriate corrections made to the database prior to the cutting of any blocks. This ensures that only eligible samples are shipped and expedites the verification and updating of the data for the applicant.

All quality control procedures described in Section 2 are adhered to. Frequent communication is maintained between IMS and the sites to obtain up-to-date information on the preparation process, and to respond to any questions or problems. IMS also keeps the applicant informed regarding the status of the slide preparation and anticipated shipment dates.

IMS flags selected specimens in the database so that they won't be accessed for other requests until the shipping site indicates the case will be available for future studies. Each site is

responsible for informing IMS about which specimens were shipped and when the shipment occurred. In the case of missing blocks or tissue, or other circumstances that result in the unavailability of specimens, the Central Data Center should be notified, and an alternate case will be selected if the replacement samples provided have been exhausted.

4.5 Shipment of Specimens

Once the specimens are ready for shipping, the data coordinator at each site cross-checks the Specimen List/Confirmation log with the slide numbers to verify that what is on the list is actually being sent. One copy is included in the shipment for verification at the receiving end, one copy is maintained at the site, and one is returned to IMS by fax or electronic mail. IMS contacts the Investigator (or designated recipient of the slides) to inform them that the slides have been shipped and to request immediate feedback regarding the receipt of the slides, timeliness of the shipment and the quality of the slides received. IMS sends a Customer Satisfaction Survey, to the Investigator within 90 days after the final shipment of all slides.

4.6 Preparation of Data Report

After the final tissue sections have been mailed, IMS begins to prepare the clinical data report for the receiving investigator. This report is created in Excel and contains only the data elements requested in the application, not all data elements for each case. Prior to generating the report, IMS also verifies that each site has submitted any recent corrections/updates to the shipped cases. The final report (Excel file) is sent to the investigator by email after payment for processing the shipments is received.

4.7 Billing

IMS queries each site for verification of the final number of cases shipped, shipping costs, and any other special fees incurred. As soon as the information is received from all sites, IMS prepares one invoice for the total cost of the panel and submits it to the applicant (Exhibit 4-5). The payment due date is 30 days from the date of the invoice.

All payments received from applicants are deposited into a non-interest bearing account for the CPCTR immediately upon receipt. Within ten days of receipt of payment, IMS sends a check to each site for their portion of funds to cover the cost of slide preparation and shipping.

IMS maintains a Shipment Summary for each approved application request. This form will be used to track invoices sent, payments received, payments disbursed, shipments, date shipped and results received.

Charges for CPCTR specimens

(Specimens will not be shipped until processing fees and shipping charges are paid)

Cases: $40 per case, which includes 4 standard 5-micron or 2, 10-micron slides (one section per slide). Extra 5-micron, slides cost $3.00 each. Extra 10-micron slides cost $5.00 each.

$50 for RNA or DNA analysis (requires a clean microtome blade for each block), which includes 4 standard 5-micron or 2, 10-micron slides (one section per slide). Extra 5-micron slides cost $4.00 each. Extra 10-micron slides cost $6.00 each.

$3.00 for standard 5-micron slide
$4.00 for 3-4-micron slides

$5.00 for 10-micron slides
$10.00 for 11-<25-micron thick section on slide
$20.00 for 25-micron or thicker section (placed in tube for PCR analysis)

$4.00 for slide with Hematoxylin & Eosin-stained section

$100 for frozen tissue specimen, not to exceed 0.2 grams

All charges will be tripled for industries requesting material.

4.8 Study Results

The resource application agreement requires investigators to report the published results of their studies to the Resource. IMS submits a follow-up letter (Attachment H) semi-annually to ask investigators about the status of their study. Reports received are filed after copies are forwarded to the NCI and the Coordinating Committee.

4.9 Applying for Tissue Microarrays from the Resource

The CPCTR application process for tissue microarray slides is simple and straightforward. The process is outlined below:

1. You submit a *Letter of Intent* (LOI) to the resource. This letter is to tell us about your research plan to help us determine whether array slides are appropriate to answer the question(s) you are posing.

2. The CPCTR Research Evaluation Panel (REP) will review your LOI as rapidly as possible. If they have any questions about your letter, we will contact you directly (via e-mail or telephone) to discuss the concerns.

3. If approval is recommended by the REP, the LOI will be reviewed by the CPCTR Coordinating Committee for merit and availability of the specific TMA.

4. You will be informed of the outcome and if approved, when you can expect to receive the slides you requested. No further application will be required.

4.10 Tissue Microarray (TMA) Agreement

CPCTR TMAs are provided as a service to the research community without warranty of merchantability or fitness for a particular purpose or any other warranty, express or implied. You must agree that tissue microarrays (TMAs) provided by the Cooperative Prostate Cancer Tissue Resource (CPCTR) will be used for the research purposes specified in your *letter of intent* or the *short form application.*

The NCI is interested in learning about the quality of the tissue microarray specimens you receive from the Cooperative Prostate Cancer Tissue Resource (CPCTR) and your satisfaction with the TMA specimens. We would greatly appreciate a quality assurance statement from you about how well your experiments worked. As an alternative to a quality assurance statement, you may send us your raw data (omitting the identity of the probe used, if so desired) and we will determine the results. This data is for NCI internal use only. The confidentiality of the data and your right to publish results will not be restricted and your data will not be shared with anyone else.

You also agree to acknowledge the contributions of the CPCTR in all publications resulting from your use of CPCTR TMA slides. Recommended wording to the methods or acknowledgment section is as follows: *Tissue microarray slides were provided by the Cooperative Prostate Cancer Tissue Resource which is funded by the National Cancer Institute. Other investigators may have received slides from these same array blocks.*

Please provide us with the citation of any publication resulting from research on CPCTR TMA slides as soon as it is available.

THE COOPERATIVE PROSTATE CANCER TISSUE RESOURCE WILL PROVIDE APPROVED SPECIMENS UPON RECEIPT OF THIS FORM BY E-MAIL TO: sherrill.long@imsweb.com

We will be happy to discuss these requirements and the timing of your submission with you prior to shipment of the specimens. Please contact Jules Berman MD (Phone 301-435-9006) e-mail bermanj@mail.nih.gov if you have any questions.

Signature of investigator: ____________________________________

4.11 Short Form Application

Application for small numbers of specimens can be sent directly to the Principal Investigator (e.g. Dr. Mike Becich) bypassing the RER process altogether.

NCI Cooperative Prostate Cancer Tissue Resource- Short Form Application

Mike Becich, M.D.
University of Pittsburgh Medical Center
Shadyside Hospital
Room # WG02.6
5230 Centre Avenue
Pittsburgh, PA 15232

Approved: ___________
Date: _______
Comments: __________
____
____

RE: Request for a small number of samples from the Cooperative Prostate Cancer

Dear Dr. Becich:

I am requesting a minimal number of samples from the CPCTR:
__ Test development __ Quality control __ Preliminary research
__ Other (specify) ____

Project/Title: ____
____

I am requesting (select one (1) only):

__ Three (3) optimization tissue microarray (TMA) slides (without annotation) of radical prostatectomies to test the probe

__ Five (5) slides (specify if they should be silanated) with 5-micron paraffin sections of cancer from 5 different radical prostatectomy specimens, specify Gleason score(s) if pertinent:
____

__ Five (5) slides (specify if they should be silanated) with 5-micron paraffin sections of high grade prostatic intraepithelial neoplasia (HGPIN) from 5 different radical prostatectomies:
____

__ Five (5) slides (specify if they should be silanated) with 5-micron sections of benign prostatic tissue from 5 different radical prostatectomies: _____________________

__ Two (2) fresh frozen tissue specimens (0.2 gms each) containing: cancer ___, HGPIN ___ or benign glands ___.

__ Three (3) 0.5 ml serum samples from patients with cancer

__ Three (3) 0.5 ml plasma samples from patients with cancer

I agree that this is a one time only request, and I understand that I am expected to complete and submit a full application to the CPCTR if I need any further samples. I certify that I have the requisite institutional approvals necessary to conduct this research. I will provide a progress report about my project to the CPCTR within 6 months after receipt of specimens along with any publications resulting from the use of these specimens. I also agree that I will cite the Cooperative Prostate Cancer Tumor Resource in any publication.

Sincerely yours,

____
Investigator's signature

____
Investigator's printed name

____
Title

____
Date

Investigator's contact information

Institution: ___________________________
Department: __________________________
Telephone:____________Fax: __________
Email: ______________________________

Mailing address: ______________________
____
____

Specimen shipping address (if different)
____
____
____

EXHIBIT 4-9
APPLICATION TRACKING FORM
(MS Excel Spreadsheet)

EXHIBIT 4-10
(CPCTR) - Shipment Invoice

**Billing Office Name & Address:**<br><br>Attention:	**Payee's Name and Address:**<br>Payable to: **CPCTR**<br>Information Management Services, Inc.<br>6110 Executive Blvd., Suite 310<br>Rockville, MD 20852<br>Attention: Sherrill Long (301) 984-3445
**Reference:** **CPCTR Application #000n**	**Investigator:**
**Date Invoice Prepared:** May 22, 1998	
**Date Invoice Due:** **June 22, 1998**	
**Submitted by:** Sherrill Long	

Description of Resource Materials Provided:

**Number of Cases**<br><br>**George Washington**<br>**Medical College of Wisconsin**<br>**New York University**<br>**University of Pittsburgh**	**Total**<br>**(#)**<br>**(#)**<br>**(#)**<br>**(#)**<br>**(#)**	**e.g. $40/case**	**$**
**Number of Sections**<br>**Cancer**<br>**Normal Tissue**<br>**Frozen tissue**<br>**PCR specimens**<br>**PBMC**<br>**Serum**	 **# of extra slides**<br>**# of extra slides**<br>**#**<br>**#**	 **e.g. $3 each**<br>**e.g. $3 each**<br>**$100 each**<br>**$50/case**	 **$**<br>**$**<br>**$**
**Subtotal**			**$**
**Shipping & Handling**			**$**
**TOTAL**			**$**

SECTION 5. MARKETING AND WORLD WIDE WEB DEVELOPMENT

5.1. Advertising methods

The CPCTR will use various media for advertising the availability of the tissues and services available to the research community. The list of methods will include, but not be restricted to:

1- *Web page with links to the, e.g., NIH, NCI, NCBI and CGAP websites:* It will be hosted at IMS as one of the pages of NCI. The website will be built and maintained by all sites, with support from the IMS and NCI, under supervision of the Marketing Subcommittee and ultimately by the Coordinating Committee of the CPCTR. The website will include general information about the CPCTR, information about the type of specimens available, a searchable database of the cases, and forms for making tissue requests and inquiries.

2- *Mass mailings to potential users:* Letters and/or e-mails will be sent to investigators that have published articles in tissue-based prostate research during the last three years. The postal and e-mail addresses are available in the address for reprint requests, cancer research societies, and the NIH CRISP database. The address of pharmaceutical companies and other industrial enterprises will be obtained by web search and other methods. The Marketing Subcommittee will write the letters, with an equal distribution by each CPCTR site. A mechanism to be deleted from any mailing list will be offered to each e-mail recipient.

3- Ads can be placed in:
   a) Specialty journal;
   b) Research societies newsletters;
   c) Fliers at research meetings; and
   d) Journal and website free listings.

4- *Word of mouth,* especially at research meetings.

5- *Posters and podium presentations at research meetings,* presenting scientific and practical aspects of the CPCTR collection.

6- *Booths at research meetings,* in conjunction with other NCI resources, or as stand-alone booths.

The four sites of the CPCTR will equally share funding for these efforts after approval by the Coordinating Committee.

5.2 Responsibility of the advertising effort:

All members of the CPCTR will share responsibility of the advertising effort. It will be the responsibility of the Marketing Subcommittee to implement any advertising effort, under supervision of the Coordinating Committee.

5.3 Market analysis:

In order to determine what type of clientele will make use of the CPCTR, it will be necessary to conduct a market analysis. This will be done by:

1- Discussing experiences of previous tissue banks;
2- Making informal calls to investigators who may use the CPTCR;
3- Discussing the needs of the research community with members of the REP; and
4- Working with the NCI to avoid duplications and to be well informed of what projects NCI is sponsoring.

5.4 Timeline:

Formal advertising of the CPCTR resource should go public when the following items are in place:

1- Two thousand prostatectomies are accrued at the CPCTR;
2- The website is already functional;
3- The REP is constituted;
4- Shipping and handling methods and costs have been established;
5- The Manual of Operations is complete;
6- The brochure and exhibits are completed (possibly with NIH help); are produced and
7- Marketing and advertising strategies are approved by the Coordinating Committee.

Informal advertising of the CPCTR can be made any time before all the above items are in place, and will consist of:

1- Word of mouth advertising; and
2- Informal presentations.

5.5 Overview of Website

Since the purpose of the CPCTR is to provide researchers with access to primary prostate cancer tissue and associated data, a major priority is to disseminate information about the availability of the Resource’s specimens and data. Advancements in technology and the

evolution of the Internet have provided a new cost-effective means of disseminating information quickly and accurately in electronic formats.

5.6 CPCTR Web Site

IMS developed, and maintains the Web site for the CPCTR. The Web site resides on the IMS Web server and is located at http://prostatetissues.org. In collaboration with the CPCTR members, IMS designed Web pages with standard graphic and hypertext capabilities. The Web site provides information on the mission of the CPCTR, participants, procedures and requirements for obtaining tissue, processing fees, links to related sites such as additional specimen resources and the NCI. It also includes the capacity to submit letters of intent electronically.

The CPCTR website will eventually includes a database with a real-time query interface that allows internet access to categorical data about the CPCTR collection. No data will be provided on individual cases.

The CPCTR web site also includes a password-protected file transfer site (http://prostatetissues.org/files). The FTP site was set up for the Data Center (IMS) to post LOIs, documentation, publications and other reports (such as data frequencies, Status of Applications, et.) for review by CBCTR members only.

5.7 Website Reporting

Web Trends software is used to provide access-log analysis for the CPCTR website. This software analyzes the log files created by the Web server and provides invaluable information on how users access the Website. Web Trends provides statistical information as well as colorful graphs that show trends, usage, and much more. Customized reports can be generated to compare specific activities among multiple time periods. These reports help to:

- Determine the impact of Web site advertising
- Measure both quality and quantity of visitors.
- Count how many users visit the site daily and find out whether that number is growing or shrinking.
- Learn which paths visitors follow when they browse our Web site.
- Find out which countries, cities, and states the users connect from.
- Determine the most active day of the week and hour of the day.

Reports are automated so that they are updated weekly, and stored in HTML on a Web server accessible to the Internet. The addresses of the reports will not be published beyond the members. The URLs will not be referred to by any publicly accessible Web sites.

RESEARCH EVALUATIONS PANEL GUIDELINES

6.1 Research Evaluation Panel (REP) Conflict of Interest Clause

The members of the Research Evaluation Panel (REP) of the NCI Cooperative Prostate Cancer Tissue Resource (CPCTR) agree to avoid conflicts of interest in the review of research projects. The NIH conflict of interest policy states that institutions receiving grant or contract support from the NIH will have a written conflict of interest statement and will notify designated institutional officials of potential conflicts of interest. There is no requirement that those conflicts be reported to the NIH if they can be resolved at the institutional level.

The REP conflict of interest policy will follow the spirit of the NIH policy. REP members will be expected to inform each other of any major commercial or personal research activities that might influence their reviews of submitted applications. Conflict of interest is defined as:

1. Any financial interest or arrangement with a company whose product is involved in or would be affected by a study under review.

2. Any financial interest or arrangement with a competing organization or research group.

3. Any other financial connections, direct or indirect, or other situations that might raise the question of bias in the conduct of reviewing a study, including personal relationships or close collaborations.

REP members are not required to reveal confidential or proprietary information, but are expected to indicate when they may be in conflict of interest. When conflicts exist, REP members will recuse themselves from the review.

6.2 Research Evaluation Panel (REP) Summary of Review Guidelines

The REP is responsible for determining the importance of the proposed studies, the areas of weakness that require improvement, and for developing the recommendation to the Coordinating Committee. The review should be scientifically rigorous, but the written review does not need to be a lengthy document. Since the written review will be primarily for internal documentation and to guide discussions with the investigators, it can even be in 'bullet' form if it makes the preparation easier.

The review is organized into three sections:

Importance What is the importance of the basic question(s) being addressed, without respect to study design problems;

Areas Requiring Improvement Identify weaknesses and list recommendations for modifications; and

Recommendation

1) the study should receive CPCTR tissue and data,
2) the study should receive tissue and data if the identified weaknesses are corrected, or
3) the study should not receive CPCTR tissue and data.

SECTION 7 THE CENTRAL DATA CENTER & COMMON DATA ELEMENTS

7.1 Data from the four member organizations is merged into a central database designed and maintained by IMS, the Central Data Center. The primary and most important purpose of the central database is to allow retrieval of the information needed to determine the availability of appropriate specimens and data for proposed research on CPCTR resources. The database contains information about the available tissue blocks and associated clinical and follow-up data.

The Data Center responsibilities as directed by the NCI are as follows:

- Development and maintenance of a central database that can be queried for specimen availability and selection.
- Tracking and responding to inquiries regarding the Resource.
- Processing of requests and coordination of applications and shipments.
- Preparation of invoices, collection of payments and distribution of payments for shipped specimens.
- Development and maintenance of systems to provide updated information to the CPCTR and the NCI.
- Report generation
- Development and maintenance of a database that the research community can query directly via Internet.

7.2 Quality Assurance (QA) Procedures for Clinical and Pathologic Data and Materials

CPCTR Principal Investigators regularly review clinical data and registered tissues to assure the continued quality of the Resource. These audits, which occur during the semi-annual meetings of the CPCTR, consist of an examination of a randomly selected batch of registry abstracts, their matched primary medical records from which the clinical data was abstracted and a review of the corresponding pathology slides, reports and paraffin blocks.

Clinical Data Audit: The large number of variables collected by the CPCTR represent a subset of the material collected by registrars from in- and out-patient medical records, pathology, radiology, radiation therapy, laboratory, etc. The audit review seeks to verify the abstracted material by perusal of the primary source of clinical information. The Central Data selects the

cases to be examined. Members of the Coordinating Committee and their associates review these cases and compare each data item against the patient's medical record. After this review, members discuss their findings in the subsequent general meeting of the Coordinating Committee and provide a report with recommendations as indicated by their findings. Each site is responsible for making any necessary corrections discovered during the audit process and submitting their corrected database to IMS.

Data Management

7.3 Overview

IMS maintains the central database for the CPCTR. The central database contains concatenated data from the four member organizations. The primary and most important purpose of the central database is to allow retrieval of the information needed to determine the availability of appropriate specimens and data for proposed research on CPCTR resources. The database contains information about the available tissue blocks and associated clinical and follow-up data.

7.4 Data Submission

The CPCTR database consists of a single front-end Access data entry system with a fixed format. It was designed to facilitate both the ability of the sites to enter and maintain the data transmission of data to IMS for entry into the central database.

The Access database has a front-end data entry system that allows entry/updating of data into the back-end Access database. Update of patient data may be by manual entry of "batch" entry depending upon the medical record system available at the participating hospitals. The data is to be submitted to IMS as an Excel file exported from the Access database. The data elements and user instruction provides the definitions required for the central database.

A random case identifier is assigned automatically to each patient accessioned to the Access central database. In order to protect the privacy of the patient, the case identifier is hidden from data entry personnel. IMS will use this identification number to match the updated database to the previous database for quality control purposes. In addition, this identification number will be used when identifying selected specimens for an approved application.

The data is to be submitted at regularly, as it becomes available, at the time a new request for tissue is filled, or more frequently if there are significant corrections and updates to be made. The data is submitted to IMS via electronic mail to Winnie Ricker at the following Internet address: ricker@ims.nci.nih.gov. Once received by IMS, the individual databases are quality controlled, merged into one database and loaded into all applicable query and reporting systems.

7.5 Updates

Each site maintains local control over the specimens and data in its collection. Each site is responsible for updating their inventory and data. This includes adding records to the database, modifying data fields, and updating the numbers of blocks available following shipments to researchers. In addition to the initial set of data submitted, each site will accession cases to the database as they become available or as directed by the Coordinating Committee.

As specimens are prepared for shipments, the database must be updated to reflect cases for which tissue is still available, cases for which the tissue has been depleted and any corrections/updates discovered during the pathologic and clinical data review. A complete updated replacement file must be submitted to IMS so that the central database may be updated. Replacement files may be submitted at any time. IMS will update the central database a minimum of two times per year and when significant changes or updates have been submitted. Updates will be performed more frequently as required.

7.6 Edit Checks

Upon receipt, IMS will screen the data for critical data items, as defined below. In addition, IMS will perform valid and cross-field edit checks on all accepted records.

7.7 Critical Data Items

Any records with missing or invalid critical data items will be rejected. The corresponding site will receive notification specifying which records were rejected and the cause for rejection. The site must complete the missing or invalid information and resubmit their data in order for the record to be accepted. The critical data items are as follows:

Field Name	Comments
Birthdate	Year must be valid and not unknown or missing.
Date of Diagnosis	Year must be valid and not unknown or missing.
Matrix Block #1	Identifying SP number from case plus associated data.
Matrix Block #2	Identifying SP number from case plus associated data.

The following may be unknown, but must not be blank:

Race
Is Residual Carcinoma Present at Prostatectomy?
Most Prominent Histologic Type of Invasive Cancer
Gleason Primary Grade
Gleason Secondary Grade
Gleason Sum Score

Nodes Examined
Nodes Positive
Distant Mets at Time of Diagnosis
pT Stage
pN Stage
pM Stage
Vital Status

7.8 Valid and Cross-field checks

Valid field checks as well as cross-field edits will be performed on the database as part of the data entry and also as part of each update cycle. Recodes for select variables are defined and generated to allow searchable parameters for open-range fields. The valid field options for each data element are defined in the CDE description. Any records with invalid or discrepant data items will be censored so they are not selected for an application request until they are resolved.

Error reports listing each error by case identifier will be generated and submitted to the respective site for resolution. Any mismatches or unresolved edit errors will be resolved with the site by submitting a corrected and updated database. If the site does not respond to their error report or submit a corrected replacement file within 60 days, IMS will resend the error report to the site and send a copy to the NCI for their information.

7.9 Quality Control

Following each update, the new database will be compared with the old for validation and quality control. All discrepancies will be reviewed by IMS to identify any unusual changes. A listing of these questionable data fields will be submitted to each site for review and verification. In addition, audits will be performed not only to verify the data that is collected at the member site but to also check this against the data in the central database. IMS will randomly select cases under the direction of the NCI statistician. Section 2 details all aspects of quality control for both the clinical and pathologic data in the CPCTR.

7.10 Searches and Queries to the Database

When the data files are received from the sites, the data is merged to create the combined central database. Public querying of the database is done through the WWW. Only categorical data is available to the public. Prior to the database being released on the WWW, a final set of control counts is checked on the WWW server version of the database after transmission in order to verify that no transmission error occurred..

7.11 Report Generation

IMS generates reports using the information contained in the database to provide the CPCTR with descriptive statistics of the central data or to verify the accuracy of data and detect any

incongruities. The frequency of reporting varies depending on the frequency of changes in the resource information. The more rapidly the resource information changes, the more frequently reports are generated. Reports are generated in electronic format for easy and quick distribution to the group, or for posting on the IMS file transfer site located on the CPCTR web site.

7.12 Standard reports

Standard reports and tables are generated from the central database following each update, prior to each meeting, at the time of fulfilling a request, or as requested by the CC or NCI. The standard reports include but are not limited to the following:

- Error Report
- Database Summary Report
- Status of LOIs/Applications

7.13 Customized Reports

Other types of reports are generated as requested for dissemination to the group or for use in marketing. Statistical reports are generated utilizing SAS and other packages depending on the requirements of the report. These might include cross-tabulations of various types or frequency distributions on selected variables.

SECTION 8 HISTOLOGY MANUAL

http://geocities.com/prostatemanual/Histomanual2.5.pdf

8.1 INTRODUCTION

This manual details the pathologic definitions needed to complete the CPCTR Pathology Common Data Elements (CDEs). The descriptions and definitions are based on the AFIP Fasicle 28, Third series: Tumors of the Prostate, Seminal Vesicles, Male Urethra, and Penis. Resource pathologists should use this manual as a guide for the assignment of Histology and Matrix CDEs for the CPCTR. Use of this manual as a guideline will ensure a standardized approach to assignment of the pathologic CDEs, and to the choice of paraffin blocks (matrix blocks) selected from submitted cases.

8.2 Table of Contents

The content of the manual follows the same order as the Histology component (CDEs) of the CPCTR database:

1. Histologic type of cancer
   - *A. Acinar carcinoma, NOS*
   - *B. Ductal carcinoma*
   - *C. Mucinous carcinoma*
   - *D. Signet ring cell carcinoma*
   - *E. Basal cell carcinoma*
   - *F. Transitional cell carcinoma*
   - *G. Undifferentiated non-small cell carcinoma*
   - *H. Sarcomatoid carcinoma*
   - *I. Large cell Neuroendocrine carcinoma*
   - *J. Small cell anaplastic carcinoma*
   - *K. Squamous cell or adenosquamous carcinoma*
   - *L. Mesenchymal tumor*
   - *M. Lymphoma*
   - *N. Other*
2. Gleason Grading of cancer
   - *A. Gleason Pattern 1*
   - *B. Gleason Pattern 2*
   - *C. Gleason Pattern 3 (a, b, c)*
   - *D. Gleason Pattern 4 (a, b)*
   - E. *Gleason Pattern 5 (a, b)*
3. Percentage of Gleason 4/5
4. Size of largest nodule of Invasive Cancer
5. Percentage of Gland occupied by tumor
6. Extraprostatic Extension / Extracapsular Invasion
7. Surgical Margin Involvement
8. High Grade Prostatic Intraepithelial Neoplasia
9. Perineural Invasion
10. Seminal Vesical Invasion
11. Angio/Lymphatic Invasion
12. Pathologic Staging
